# Midlife occupational cognitive requirements protect cognitive function in old age by increasing cognitive reserve

**DOI:** 10.3389/fpsyg.2022.957308

**Published:** 2022-12-08

**Authors:** Luca Kleineidam, Steffen Wolfsgruber, Anne-Sophie Weyrauch, Linn E. Zulka, Simon Forstmeier, Sandra Roeske, Hendrik van den Bussche, Hanna Kaduszkiewicz, Birgitt Wiese, Siegfried Weyerer, Jochen Werle, Angela Fuchs, Michael Pentzek, Christian Brettschneider, Hans-Helmut König, Dagmar Weeg, Horst Bickel, Melanie Luppa, Francisca S. Rodriguez, Silka Dawn Freiesleben, Selin Erdogan, Chantal Unterfeld, Oliver Peters, Eike J. Spruth, Slawek Altenstein, Andrea Lohse, Josef Priller, Klaus Fliessbach, Xenia Kobeleva, Anja Schneider, Claudia Bartels, Björn H. Schott, Jens Wiltfang, Franziska Maier, Wenzel Glanz, Enise I. Incesoy, Michaela Butryn, Emrah Düzel, Katharina Buerger, Daniel Janowitz, Michael Ewers, Boris-Stephan Rauchmann, Robert Perneczky, Ingo Kilimann, Doreen Görß, Stefan Teipel, Christoph Laske, Matthias H. J. Munk, Annika Spottke, Nina Roy, Frederic Brosseron, Michael T. Heneka, Alfredo Ramirez, Renat Yakupov, Martin Scherer, Wolfgang Maier, Frank Jessen, Steffi G. Riedel-Heller, Michael Wagner

**Affiliations:** ^1^Department of Neurodegenerative Diseases and Geriatric Psychiatry, University Hospital Bonn, Bonn, Germany; ^2^German Center for Neurodegenerative Diseases (DZNE), Bonn, Germany; ^3^Department of Psychology and Centre for Ageing and Health (AgeCap), University of Gothenburg, Gothenburg, Sweden; ^4^Developmental Psychology and Clinical Psychology of the Lifespan, University of Siegen, Siegen, Germany; ^5^Department of Primary Medical Care, Center for Psychosocial Medicine, University Medical Center Hamburg-Eppendorf, Hamburg, Germany; ^6^Medical Faculty, Institute of General Practice, University of Kiel, Kiel, Germany; ^7^Center for Information Management, Hannover Medical School, Hanover, Germany; ^8^Medical Faculty, Central Institute of Mental Health, Mannheim/Heidelberg University, Heidelberg, Germany; ^9^Medical Faculty, Centre for Health and Society (CHS), Institute of General Practice (ifam), Heinrich Heine University, Düsseldorf, Germany; ^10^Department of Health Economics and Health Services Research, Hamburg Center for Health Economics, University Medical Center Hamburg-Eppendorf, Hamburg, Germany; ^11^Department of Psychiatry and Psychotherapy, School of Medicine, Technical University of Munich, Munich, Germany; ^12^Medical Faculty, Institute of Social Medicine, Occupational Health and Public Health (ISAP), University of Leipzig, Leipzig, Germany; ^13^German Center for Neurodegenerative Diseases (DZNE), Berlin, Germany; ^14^Department of Psychiatry, Campus Berlin-Buch, Charité-Universitätsmedizin Berlin, Corporate Member of Freie Universität Berlin, Humboldt-Universität zu Berlin, Berlin Institute of Health (BIH), Berlin, Germany; ^15^Memory Clinic and Dementia Prevention Center, Experimental and Clinical Research Center (ECRC), Berlin, Germany; ^16^Department of Psychiatry, Campus Benjamin Franklin, Charité—Universitätsmedizin Berlin, Corporate Member of Freie Universität Berlin, Humboldt-Universität zu Berlin, Berlin Institute of Health (BIH), Berlin, Germany; ^17^Department of Psychiatry and Psychotherapy, Charité—Universitätsmedizin Berlin, Berlin, Germany; ^18^University of Edinburgh and UK DRI, Edinburgh, United Kingdom; ^19^Department of Psychiatry and Psychotherapy, University Medical Center Goettingen, University of Goettingen, Goettingen, Germany; ^20^German Center for Neurodegenerative Diseases (DZNE), Goettingen, Germany; ^21^Leibniz Institute for Neurobiology, Magdeburg, Germany; ^22^Department of Medical Sciences, Neurosciences and Signaling Group, Institute of Biomedicine (iBiMED), University of Aveiro, Aveiro, Portugal; ^23^Department of Psychiatry, Medical Faculty, University of Cologne, Cologne, Germany; ^24^German Center for Neurodegenerative Diseases (DZNE), Magdeburg, Germany; ^25^Institute of Cognitive Neurology and Dementia Research (IKND), Otto-von-Guericke University, Magdeburg, Germany; ^26^German Center for Neurodegenerative Diseases (DZNE), Munich, Germany; ^27^Institute for Stroke and Dementia Research (ISD), University Hospital, LMU Munich, Munich, Germany; ^28^Department of Psychiatry and Psychotherapy, University Hospital, LMU Munich, Munich, Germany; ^29^Munich Cluster for Systems Neurology (SyNergy), Munich, Germany; ^30^Ageing Epidemiology Research Unit (AGE), School of Public Health, Imperial College London, London, United Kingdom; ^31^Sheeld Institute for Translational Neuroscience (SITraN), University of Sheeld, Sheeld, United Kingdom; ^32^German Center for Neurodegenerative Diseases (DZNE), Rostock, Germany; ^33^Department of Psychosomatic Medicine, Rostock University Medical Center, Rostock, Germany; ^34^German Center for Neurodegenerative Diseases (DZNE), Tübingen, Germany; ^35^Section for Dementia Research, Hertie Institute for Clinical Brain Research and Department of Psychiatry and Psychotherapy, University of Tübingen, Tübingen, Germany; ^36^Department of Biology, Technische Universität Darmstadt, Darmstadt, Germany; ^37^Department of Neurology, University of Bonn, Bonn, Germany; ^38^Excellence Cluster on Cellular Stress Responses in Aging-Associated Diseases (CECAD), University of Cologne, Cologne, Germany; ^39^Division of Neurogenetics and Molecular Psychiatry, Department of Psychiatry and Psychotherapy, Faculty of Medicine, University Hospital Cologne, University of Cologne, Cologne, Germany; ^40^Department of Psychiatry and Glenn Biggs Institute for Alzheimer's and Neurodegenerative Diseases, San Antonio, TX, United States

**Keywords:** cognitive reserve, brain maintenance, brain reserve, mid-life cognitive demands, Alzheimer's disease, occupation

## Abstract

**Introduction:**

Several lifestyle factors promote protection against Alzheimer's disease (AD) throughout a person's lifespan. Although such protective effects have been described for occupational cognitive requirements (OCR) in midlife, it is currently unknown whether they are conveyed by brain maintenance (BM), brain reserve (BR), or cognitive reserve (CR) or a combination of them.

**Methods:**

We systematically derived hypotheses for these resilience concepts and tested them in the population-based AgeCoDe cohort and memory clinic-based AD high-risk DELCODE study. The OCR score (OCRS) was measured using job activities based on the O*NET occupational classification system. Four sets of analyses were conducted: (1) the interaction of OCR and APOE-ε4 with regard to cognitive decline (N = 2,369, AgeCoDe), (2) association with differentially shaped retrospective trajectories before the onset of dementia of the Alzheimer's type (DAT; N = 474, AgeCoDe), (3) cross-sectional interaction of the OCR and cerebrospinal fluid (CSF) AD biomarkers and brain structural measures regarding memory function (N = 873, DELCODE), and (4) cross-sectional and longitudinal association of OCR with CSF AD biomarkers and brain structural measures (N = 873, DELCODE).

**Results:**

Regarding (1), higher OCRS was associated with a reduced association of APOE-ε4 with cognitive decline (mean follow-up = 6.03 years), consistent with CR and BR. Regarding (2), high OCRS was associated with a later onset but subsequently stronger cognitive decline in individuals converting to DAT, consistent with CR. Regarding (3), higher OCRS was associated with a weaker association of the CSF Aβ42/40 ratio and hippocampal volume with memory function, consistent with CR. Regarding (4), OCR was not associated with the levels or changes in CSF AD biomarkers (mean follow-up = 2.61 years). We found a cross-sectional, age-independent association of OCRS with some MRI markers, but no association with 1-year-change. OCR was not associated with the intracranial volume. These results are not completely consistent with those of BR or BM.

**Discussion:**

Our results support the link between OCR and CR. Promoting and seeking complex and stimulating work conditions in midlife could therefore contribute to increased resistance to pathologies in old age and might complement prevention measures aimed at reducing pathology.

## Introduction

The occurrence of dementia in old age is not inevitable. Even in the highest age groups, some individuals show only limited neurodegeneration (Braak et al., 2011), whereas others show only minor cognitive deficits in the presence of substantial neuropathological changes (Katzman et al., 1988; Azarpazhooh et al., 2020). This phenomenon has often been linked to concepts, such as cognitive reserve and its popular proxy measure of education (Stern, 2012; Stern et al., 2020). Higher education is associated with a reduced risk of dementia (Meng and D'Arcy, 2012) and has been shown to mitigate the effects of pathology on cognitive functions (Brayne et al., 2010; Wolf et al., 2019; Zahodne et al., 2019; Joannette et al., 2020; Soldan et al., 2020).

Importantly, cognitive activities beyond childhood and young adulthood, such as occupational cognitive activities in midlife (Kröger et al., 2008; Smart et al., 2014; Pool et al., 2016; Then et al., 2017), also provide protection against dementia (even when adjusting education). Midlife activities mediate parts of the protective association of education (Fujishiro et al., 2019), stressing the potential of continuing cognitive activities. Recently, Pool et al. (2016) proposed the “occupational cognitive requirements score” (OCRS) as a global indicator that still precisely reflects the fine-grained interindividual differences in cognitive activity levels associated with one's occupation. It is based on the Department of Labor's Occupational Information Network (O^*^NET) database (http://www.onetonline.org) that contains a detailed description of job characteristics and requirements. To derive the OCRS, only a job title (and a description of performed task where appropriate) is needed to map jobs to the O^*^NET database and score the occupational cognitive activities on a continuous scale. Pool et al. (2016) showed that the OCRS is related to slower cognitive decline in old age, but OCRS did not significantly interact with carrying an APOE-ε4 allele with respect to cognitive decline. Participants in this study were aged 65 years and above at baseline, were not selected based on cognitive status, and were followed up for 8 years on average.

The OCRS offers an another way to study the protective role of midlife occupational cognitive activities in cognitive decline and dementia based on occupational complexity (Kröger et al., 2008; Smart et al., 2014; Boots et al., 2015). While both occupational complexity and the OCRS measure to some degree the work-related cognitive demands, the OCRS assesses more directly the actual level of performed occupational cognitive activities.

### Aims and research approach

In our study, we replicated the analysis of Pool et al. and extended it further, as explained in the following sections.

**Figure 1:**
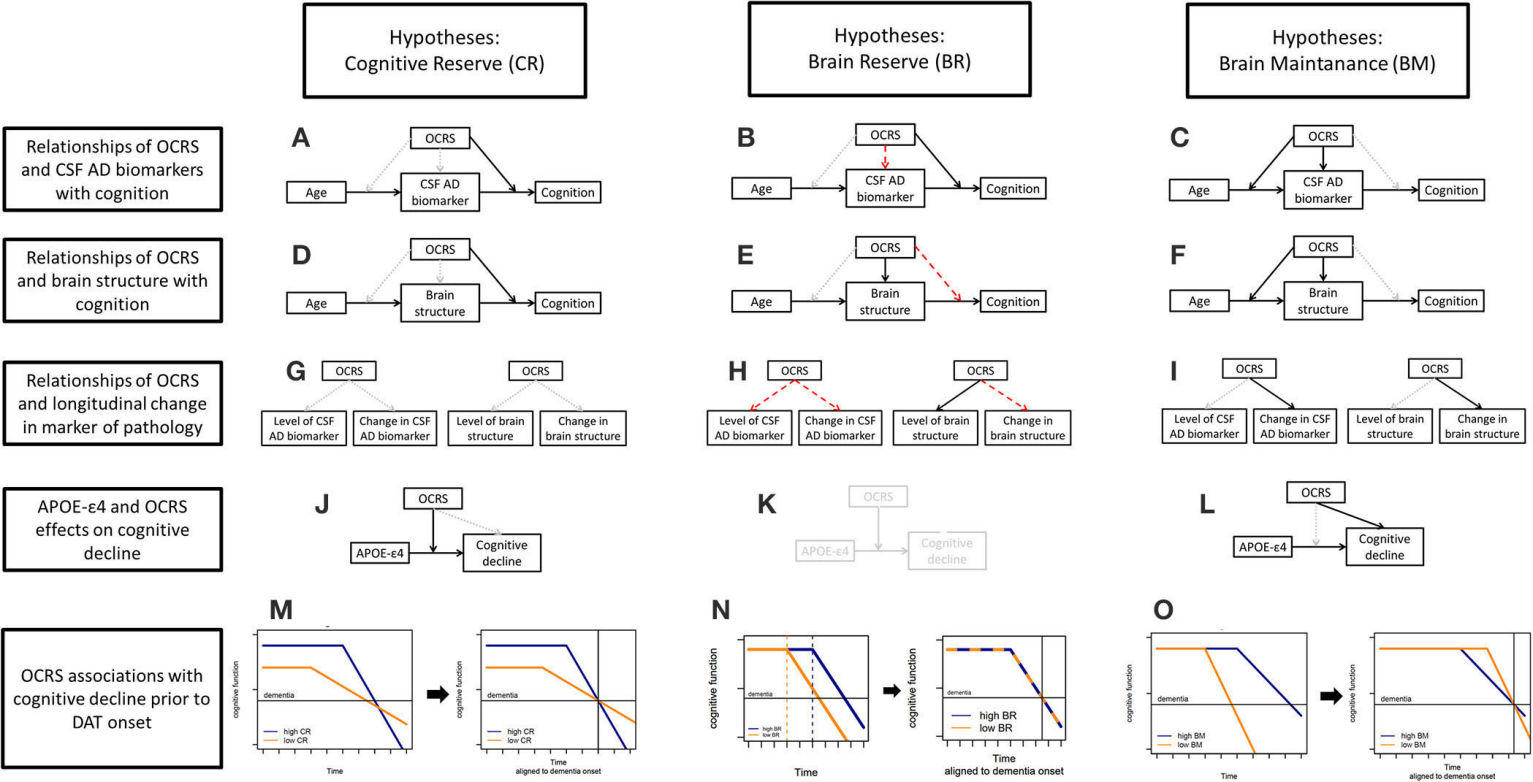
**(A)** Hypotheses for CR regarding the relationship of the OCRS and CSF AD biomarkers with cognition. **(B)** Hypotheses for BR regarding the relationship of the OCRS and CSF AD biomarkers with cognition. **(C)** Hypotheses for BM regarding the relationship of the OCRS w OCRS and CSF AD biomarkers with cognition. **(D)** Hypotheses for CR regarding the relationship of the OCRS and brain structure with cognition. **(E)** Hypotheses for BR regarding the relationship of the OCRS and brain structure with cognition. **(F)** Hypotheses for BM regarding the relationship of the OCRS and brain structure with cognition. **(G)** Hypotheses for CR regarding the relationship of the OCRS with longitudinal change in markers of pathology. **(H)** Hypotheses for BR regarding the relationship of the OCRS with longitudinal change in markers of pathology. **(I)** Hypotheses for BM regarding the relationship of the OCRS with longitudinal change in markers of pathology. **(J)** Hypotheses for CR regarding the relationship of the OCRS and APOE with cognitive decline in general population-based cohorts. **(K)** Hypotheses for BR regarding the relationship of the OCRS and APOE with cognitive decline in general population-based cohorts. **(L)** Hypotheses for BM regarding the relationship of the OCRS and APOE with cognitive decline in general population-based cohorts. **(M)** Hypotheses for CR regarding the relationship of the OCRS with cognitive decline prior to the onset of dementia of the Alzheimer's type (DAT). **(N)** Hypotheses for BR regarding the relationship of the OCRS with cognitive decline prior to the onset of dementia of the Alzheimer's type (DAT). **(O)** Hypotheses for BM regarding the relationship of the OCRS with cognitive decline prior to the onset of dementia of the Alzheimer's type (DAT). OCRS, occupational cognitive requirement score; APOE−+4, apolipoprotein E +4 allele; CSF, cerebrospinal fluid; CR, cognitive reserve; BM, brain maintenance; BR, brain reserve.

We aimed to extend previous research by exploring the specific resilience mechanisms that may convey the protective role of OCRS in cognitive decline. Research on reserve and resilience has developed definitions of three concepts that explain interindividual differences in the development of pathologies and their impact on cognitive function: cognitive reserve (CR), brain maintenance (BM), and brain reserve (BR). In this study, we refer to the definitions proposed by Stern et al. (2020), which are largely consistent with more recent definitions by the Collaboratory on Research Definitions for Reserve Resilience in Cognitive Aging Dementia (2022). However, we acknowledge that some differences in the definitions proposed by other authors may exist (Cabeza et al., 2018; Ewers, 2020). To assess the link between the OCRS and resilience concepts, we derived a set of hypotheses on expected associations of the OCRS with different outcomes in a population-based and memory clinic-based cohort. All hypotheses are graphically summarized in Figure 1. Of note, the hypotheses displayed in Figure 1 illustrate expected relationships in case the OCRS would solely act through the respective resilience concept. The empirical pattern of the results is then compared to these expectations.

We derived hypotheses for three different settings: (a) available direct measures of pathology and cognition in a memory clinic cohort; (b) no data on directly assessed pathologic markers but information on a genetic risk factor for pathology and cognitive decline in the general population; and (c) information only on cognitive trajectories in individuals developing dementia of the Alzheimer's type (DAT). For the latter case, we systematically derived the expected trajectories of the development of pathology and cognitive function for individuals with either high or low resilience in each concept (Supplementary Figure 1). To specify hypotheses on the expected cognitive trajectories aligned to the onset of DAT, the derived prototypical cognitive trajectories were then moved graphically along the x-axis (time) until both trajectories aligned at a hypothetical dementia onset (Supplementary Figure 1, Figures 1M–O). We also discussed the caveats for interpretation in each setting with regard to the operational definitions proposed in the literature.

### Hypotheses on the link of the OCRS with CR

#### Definition of CR

In our study, we defined CR as the brain's ability to actively adapt to the presence of pathologies and mitigate their impact on cognition, leading to higher cognitive functioning than expected based on pathologic brain changes (Stern et al., 2020). Historically, it has been assumed that the adaption of cognitive processes can compensate pathologies up to a “point of inflection” after which individuals with high reserve should show an accelerated decline in cognitive functioning (Stern, 2012). We consider this phenomenon to be a characteristic of CR. Furthermore, CR has been operationally defined as an amelioration of the effect of pathology on cognition; accordingly, a high level of a CR marker should relate to a weaker association between a measure of brain pathology and neuropsychological test performance (Stern et al., 2020).

#### Hypotheses and empirical tests of CR using direct assessments of pathology

First, we used data from the memory clinic-based German Center for Neurodegenerative Diseases (DZNE) Longitudinal Cognitive Impairment and Dementia Study (DELCODE) (Jessen et al., 2018), providing direct assessments of pathology and cognitive function in a population at increased risk for Alzheimer's disease (AD). We focused on two groups of pathology markers: (1) cerebrospinal fluid (CSF) biomarkers indexing AD pathologic changes (i.e., CSF Abeta42/40 and pTau181) since DELCODE is enriched for individuals at risk for AD (see Methods), and (2) measures of brain structure integrity such as hippocampal volume and temporal cortex thickness obtained from MRI scans, as those brain regions are especially vulnerable to AD and age-related pathologic changes (Fjell et al., 2013), and global measures of brain structure and neuronal loss (i.e., total gray matter volume and CSF total tau). Regarding cognition, we focused on memory function, which is the most severely affected cognitive domain in AD, linked to AD and other neuropathologies (Wilson et al., 2019a), and is closely related to the integrity of the hippocampus and temporal lobe structures (Deweer et al., 1995). In addition, we assessed global cognitive functioning to examine the consistency of the findings when including data from other cognitive domains. Therefore, this cohort provided data to perform a recommended test for CR (Stern et al., 2020). If the OCRS mainly acts through CR, higher OCRS should be associated with a reduced association (i.e., a statistical interaction effect) of markers of AD pathology (Figure 1A), as well as markers of brain integrity with cognitive performance (Figure 1D).

#### Hypotheses and empirical tests of CR using genetic markers

Second, we aimed to assess the association between OCRS and cognitive decline in a population-based cohort of elderly individuals without dementia at baseline. This population-based cohort may allow for better generalizability of the results on OCRS, although a direct assessment of pathology is lacking. However, APOE-ε4, a strong genetic risk factor for AD (Genin et al., 2011), can serve as a proxy for a higher risk of pathologic development. Using such a risk factor for pathology is less precise than using a direct measure and can thus only provide putative evidence for a link between OCR and CR. Considering this limitation, it can be predicted that if the OCRS mainly acts through CR, higher OCRS should be associated with a reduced association of APOE-ε4 with cognitive decline, since the impact on pathologic changes should be mitigated in individuals with high CR. Statistically, this would be represented by a statistical interaction between APOE-ε4 and OCRS regarding cognitive decline (Figure 1J).

#### Hypotheses and empirical tests of CR using cognitive trajectories aligned to dementia onset

Third, we aimed to derive hypotheses on the link between OCR and CR in longitudinal cohorts without a direct assessment of pathologies or genetic risk markers as proxies. Notably, this can provide only low-level evidence for a link to CR compared to the empirical tests, including directly measured pathology. In this setting, we propose that the trajectory of cognition before and after the onset of dementia in individuals developing DAT should be examined. Importantly, all individuals who progressed to DAT developed some pathology. Therefore, assessing the trajectory of cognition in these individuals allows for the study of the adaptation of the brain to the progressive development of pathology. For individuals with a high CR, the predicted cognitive trajectories with progressively developing pathologies have been well-described (Stern, 2012). Herein, high CR should generally relate to an initially higher cognitive level and a later onset of cognitive decline (from individual-specific, previously stable levels), but a stronger cognitive deterioration afterward (Figure 1M, left plot). A stronger decline after symptom onset is expected due to the larger amount of pathology accumulated before the onset of pathology. When aligning these trajectories graphically to the onset of dementia (Figure 1M, right plot), a later onset, afterward, a stronger cognitive decline is expected for individuals with high CR. Interestingly, such a trajectory has already been demonstrated for individuals with higher education, a well-known proxy for CR (Amieva et al., 2014).

We additionally assessed whether there was evidence for a link between the OCRS and two other resilience concepts, BR and BM. In the following paragraphs, we provide definitions and empirical tests of the link between these concepts and how they relate to tests of the link to CR.

### Hypotheses on the link of the OCRS with BR

#### Definition of BR

In line with previous definitions (Stern et al., 2020), we conceptualized BR as the fixed neurobiological capital at a given time point that might have been built up during development and is passively reduced in old age with increasing pathology. Herein, BR is the quantity of neurobiological capital available at that point in time and does not include any processes related to interindividual differences in changes in brain integrity (i.e., BM, see Section Hypotheses on the link of the OCRS with BM). More available resources (i.e., higher BR) can increase the threshold for pathology that does not affect cognitive function and, therefore, delay the onset of impairments (Stern et al., 2020).

BR requires a link between certain brain features (as an indication of neurobiological capital) and cognitive function. Intracranial volume has historically been used as a proxy for BR (Stern et al., 2020) because it relates to the fact that premorbid neurobiological capital is not affected by pathologic changes. Since high BR may increase the threshold to passively tolerate pathology, it operationally relates to individual differences in cognitive function and the risk of decline at a given level of pathology (Stern et al., 2020).

#### Hypotheses and empirical tests of BR using direct assessments of pathology

First, in the memory clinic-based sample, we assessed the association of the OCRS with markers of brain structure that are related to cognitive function (i.e., hippocampal volume, temporal cortex thickness, and total gray matter volume). If OCRS acts through BR, there should be a cross-sectional association with these markers (Figure 1E). In contrast, if the OCRS mainly acts through BR, it should not relate to any longitudinal changes in brain markers in old age, as those changes are attributed to a different resilience concept [i.e., BM, see Section Hypotheses on the link of the OCRS with BM (Stern et al., 2020)]. We, therefore, examined the association of OCRS with changes in brain markers over a 1-year follow-up. If OCRS acts through BR, there should be no association with longitudinal changes in the markers of brain structure or pathology (Figure 1H). In addition, we tested the expected positive association of OCRS with intracranial volume, a proxy marker of BR that is not affected by pathology-related brain changes. Furthermore, if OCRS acts through BR, it should not be associated with cross-sectional levels or longitudinal changes in AD biomarkers since BR would not predict a direct effect on the development of neurodegenerative pathologies (Figures 1B,H; Stern et al., 2020). Notably, the link between OCRS and markers of neurodegenerative pathology and brain integrity is not part of the CR concept (Figure 1G) bearing the possibility that CR and BR may act at the same time. Therefore, the hypotheses described above focus on an additional aspect regarding the possible mechanism underlying the association of OCRS with reduced risk of cognitive decline.

Similar to CR, BR can influence the association between pathologic changes and cognition (Stern et al., 2020). However, the suspected mechanism may differ from that of CR. A high BR would result from high neurobiological resources that may passively buffer the impact of pathology on cognition until the depletion threshold of these resources. In contrast, CR is perceived as an active adaptation of cognitive processes to pathology, leading to the maintenance of high cognitive function. Both the proposed mechanisms can result in a reduced effect of pathologic alterations of proteins in the brain on cognition in cross-sectional data. Thus, if the OCRS acts through BR, a higher OCRS should be associated with a reduced association of AD biomarkers with cognition due to the buffering effect of higher neurobiological resources (Figure 1B). Operationally, this would manifest as a link of the OCRS with a measure of neurobiological resources and a reduced association of AD biomarkers with cognition that is attributable to these neurobiological resources. In contrast, BR would not be expected to modify the association between measures of brain integrity and cognition. This is because BR's protective effects should be derived from the brain structure itself and, consequently, once the structure is lost, its protective effect should be lost as well (Figure 1E). Importantly, a reduced association between markers of brain structural integrity and cognition is expected in individuals with high CR (Figure 1D). Thus, the differential predictions of BR and CR concepts regarding the reduction in the effect of brain integrity on cognitive function could provide suggestive evidence for a distinction between the two concepts. However, we emphasize that the results of these indirect tests cannot provide definite evidence. To this end, further confirmation by more direct assessments of neural mechanisms is needed (e.g., based on functional MRI). This could determine whether an active adaptation of cognitive processes or a passively increased threshold to tolerate pathology underlies this protective association. However, these assessments were not available in this study.

#### Hypotheses and empirical tests of BR using genetic markers

When examining the association of OCRS with cognitive decline in the general population depending on APOE-ε4, it is not possible to derive an empirical test of a link to BR due to the lack of a direct measure of neurobiological resources. The only possibility would be to examine the association of APOE-ε4 with cognitive decline. If the OCRS is linked to BR, one could speculate that higher OCRS could be associated with a reduced association of APOE-ε4 with cognitive decline due to a better passive tolerance of pathology due to higher neurobiological capital (Figure 1K). However, in the absence of a direct measure of neurobiological resources, any result cannot be unambiguously interpreted. For instance, CR and BR concepts would make identical predictions about the association of APOE-ε4 with cognitive decline, despite different underlying mechanisms.

#### Hypotheses and empirical tests of BR using cognitive trajectories aligned to dementia onset

In contrast, when studying the cognitive trajectories aligned with dementia onset in individuals developing an incident DAT, predictions derived from CR and BR concepts differ. During aging, high BR should relate to a later onset of dementia due to initially higher neurobiological resources. Rates of cognitive decline after the onset of deterioration from previously stable levels of cognition should develop at equal rates because BR acts through the passive increase of a threshold to tolerate pathology without any modification of the accumulation of or adaption to pathology (Figure 1N, left plot). Thus, when aligning those trajectories for dementia onset (Figure 1N, right plot), one would expect a complete alignment of cognitive trajectories and therefore no differences depending on BR. We acknowledge that the analysis only provides an indirect test of resilience mechanisms. Thus, it can only hint at the most likely underlying concept.

### Hypotheses on the link of the OCRS with BM

#### Definition of BM

BM is defined as a characteristic of the brain that accumulates fewer age-related pathologies over time and maintains high levels of functional and structural integrity in old age that accounts for cognitive performance within aging and disease (Stern et al., 2020). Therefore, BM involves a link between longitudinal changes in markers of pathology and brain integrity and cognitive function and decline. A factor related to interindividual differences in BM should be related to a reduced change in pathology and brain structure over time.

#### Hypotheses and empirical tests of BM using direct assessments of pathology

In our memory clinic sample, we investigated changes in the markers of AD pathologic changes and brain integrity during follow-up. If the OCRS mainly acts through the BM, then the high OCRS should be associated with a lower rate of change in all examined markers because a higher BM should result in a reduced accumulation rate of pathologies (Figure 1I).

In addition, it can be hypothesized that if the OCRS mainly acts through BM, then higher OCRS should relate to lower levels of pathology at baseline, since a lower rate of accumulation should have already affected the development of pathologic markers prior to the baseline assessment in our memory clinic sample. Thus, longitudinal changes in pathology before study entry should be reflected in cross-sectional measurements. Since the BR concept also assumes higher levels of neurological capital and more preserved brain integrity, cross-sectional markers of brain structure *per se* cannot differentiate between the concepts. However, some suggestive indications for the differentiation between BM and BR can be derived using this type of data. Notably, cross-sectional differences in markers of AD pathologic change are not in line with BR but, in contrast, are predicted by BM (Figures 1B,C). In addition, if reduced age-related pathology due to BM is the underlying cause of cross-sectional differences in brain structure (and AD pathologic markers), then it can be expected that the association of age with these markers should be weaker in individuals with higher OCRS (Figures 1C,F; Steffener et al., 2014). This can be hypothesized because high BM is related to a reduced age-related accumulation of pathologies, and therefore, at higher levels of the BM, age should show a less pronounced association with pathology. However, the presence of a weaker association between age and pathologic markers in individuals with high OCRS does not imply that those must necessarily derive from BM since comparisons of individuals at different ages are not identical to the assessment of actual longitudinal changes. Cross-sectional comparisons can be affected by survival bias, since older age groups cannot include individuals who died at a younger age before the assessment. Therefore, these tests can only provide indications suggestive of BM. Nevertheless, in our study, we conducted these tests to make use of larger sample sizes of cross-sectional data.

#### Hypotheses and empirical tests of BM using genetic markers

With regard to data from general population-based cohorts with information on genetic markers only, it is not possible to directly assess the link between OCRS and BM due to the lack of a direct assessment of the pathology. However, in BM concepts, it is assumed that interindividual differences in the accumulation of pathology result in differences in the development of cognition. Therefore, in a general population based study, a higher OCRS should be associated with less cognitive decline if OCRS acts through BM (Figure 1J). Reduced cognitive decline in the general population-based study could derive from a later onset of pathology or a generally lower rate of accumulation. It is important to note that the presence of this association cannot demonstrate that OCRS is specifically related to BM, since lower rates of cognitive decline can have causes besides BM, including BR and CR. However, the absence of clear evidence for this association would argue against a link between the OCRS and BM because, in this case, the association would be expected.

#### Hypotheses and empirical tests of BM using cognitive trajectories aligned to dementia onset

When examining the trajectories of cognition aligned with dementia onset, it is again not possible to derive a direct test of a link between the OCRS and BM. However, the expected shape of the trajectory when the OCRS acts through BM differs from the respective expectations derived from CR and BR. A high BM should be related to a later onset of cognitive decline from previously stable levels of cognitive performance due to later onset and/or lower rate of the development of age-related pathologies. When focusing on individuals who develop dementia despite high BM, it is expected that those individuals will still show a slower rate of accumulation of age-related pathologies, leading to a longer-lasting and more gradual development of cognitive deterioration prior to dementia as compared to individuals with low BM (Figure 1O, left plot). Therefore, when aligning expected trajectories to dementia onset (Figure 1O, right plot), high BM should relate to an earlier onset (i.e., larger temporal distance between decline onset and dementia conversion due to more gradual accumulation of pathology prior to dementia) with a slower deterioration afterward. If BM manifests solely by a delayed onset of the accumulation of pathology but thereafter similar rates, then the OCRS should not relate to any interindividual differences in cognitive trajectories aligned to dementia onset if it acts mainly through BM. In this case, predictions do not differ from those derived for BR (see Section Hypotheses and empirical tests of BR using cognitive trajectories aligned to dementia onset).

## Methods

To test the full set of hypotheses, we used data from two multicenter German cohorts: the German Study on Aging, Cognition, and Dementia in Primary Care Patients (AgeCoDe) and the DELCODE cohort.

### Sample description

#### AgeCoDe

**Figure 2:**
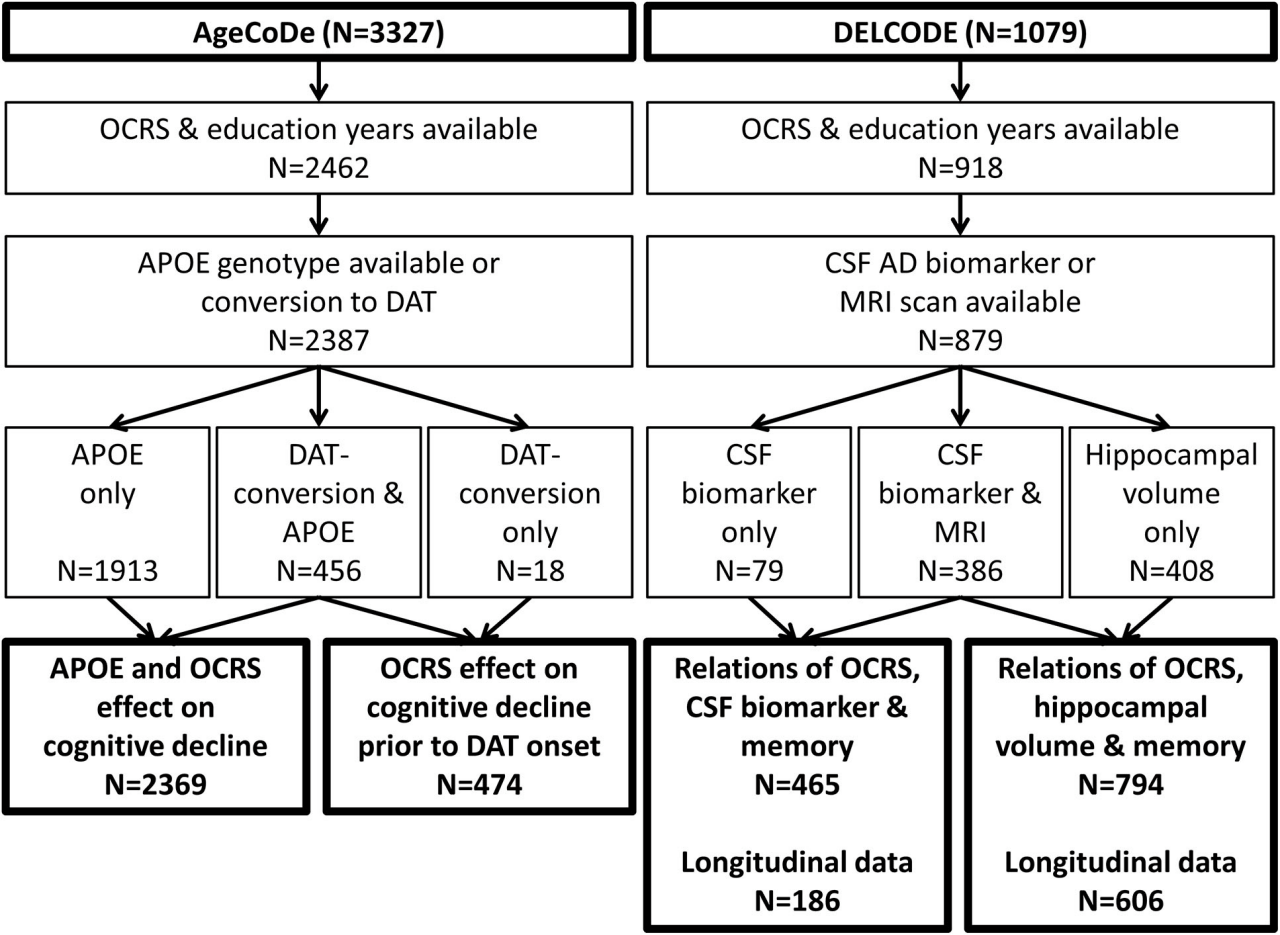
Flowchart of sample selection. APOE, apolipoprotein E; CSF, cerebrospinal fluid; DAT, dementia of the Alzheimer's type; OCRS, occupational cognitive requirement score.

We used data from 2,462 participants from the longitudinal, multicenter, prospective AgeCoDe study (Luck et al., 2007; Jessen et al., 2010). The study included randomly selected patients from 138 general practitioner (GP) practices in six German cities who were free of dementia and aged over 75 years at baseline. Exclusion criteria were consultations only by home visits, residence in a nursing home, severe illness the GP would deem fatal within 3 months, insufficient German language capabilities, deafness or blindness, inability to consent, and not being a regular patient of the participating practice. Among the 6,619 participants who could be successfully contacted, 3,327 provided informed consent and were included in the AgeCoDe cohort. The baseline examination took place between 2003 and 2004, with follow-up examinations every 18 months until 2015 (FU1–FU6) and three additional follow-ups every 10 months (FU7–FU9) in the so-called study on needs, health service use, costs, and health-related quality of life in a large sample of oldest-old primary care patients (85+) (AgeQualiDe). Questionnaires and neuropsychological assessments, including the Mini-Mental-State-Examination (MMSE) (Folstein et al., 1983), were administered at each visit. Dementia diagnoses were determined based on the respective assessments in a consensus conference with the interviewer and an experienced geriatrician or geriatric psychiatrist at each visit. Dementia diagnosis was established according to the DSM-IV criteria based on the SIDAM interview (Zaudig et al., 1991). The interview assesses cognition (55-item cognitive test battery) and impairments in activities of daily living (14-item SIDAM-ADL) and includes the Hachinski–Rosen Scale (Rosen et al., 1980). Dementia diagnosis was based on the Global Deterioration Scale (≥4) (Reisberg et al., 1982), if participants could not be personally interviewed. If sufficient information was provided, a clinical diagnosis of DAT was established according to the NINCDS-ADRDA criteria (McKhann et al., 1984). For the current analysis on cognitive trajectories prior to DAT onset, DAT participants without cerebrovascular events and those with events without temporal relationship to cognitive decline (i.e., mixed dementia) were considered. In AgeCoDe, occupational information (occupational title of the first job, last job, and longest-held job) was assessed at the second follow-up assessment. Based on this information, the OCRS was computed for 2,462 participants. Among those, 2,387 participants had information on the APOE genotype available or progressed to DAT during follow-up and could therefore be included in the current analysis (Figure 2).

All participants provided written informed consent prior to inclusion in the study. The study was approved by the ethics committee of all participating sites and conducted in accordance with the guidelines of the Declaration of Helsinki.

#### DELCODE

DELCODE (Jessen et al., 2018) is an observational, longitudinal, multicenter study conducted at 10 university-based memory clinics. The inclusion criteria were age ≥ 60 years, fluent German language skills, capacity to provide informed consent, and the presence of a study partner. Exclusion criteria were conditions clearly interfering with participation in the study or the study procedures, including significant sensory impairment, presence of specific medical conditions, or intake of specific psychoactive or anti-dementia drugs, as listed in Supplementary Text 1. DELCODE recruited patients with subjective cognitive decline (SCD), amnestic mild cognitive impairment (MCI), or DAT, who were referred to participating memory clinics. SCD patients had to report subjectively perceived cognitive decline causing concerns to the physician of the memory center and absence of a cognitive impairment defined as performance below −1.5 standard deviations (SD) in age, sex, and education-adjusted norms of the CERAD neuropsychological battery (Thalmann et al., 2000). MCI patients had to show at least a cognitive performance below −1.5 SD on the CERAD word-list delayed recall task. Patients with DAT had to show an MMSE score ≥18 and fulfilled the clinical NINCDS-ADRDA criteria (McKhann et al., 1984). In addition, a cognitively normal control group and cognitively normal first-degree relatives of patients with DAT were recruited *via* newspaper advertisements. SCD with concerns was used as an additional exclusion criterion for the control group. Detailed clinical and neuropsychological assessments and questionnaires, including an assessment of occupational information, were administered at baseline. In addition, MRI of the brain was performed, and CSF samples were collected from a subset of the participants (Figure 2). The participants were followed longitudinally during annual follow-up visits.

All participants provided written informed consent prior to inclusion in the study. The study was approved by the ethics committee of all participating sites and conducted in accordance with the guidelines of the Declaration of Helsinki.

### Assessment of the OCRS

To assess the OCRS in AgeCoDe and DELCODE, information on the job title of the participant's longest-held occupation was assessed, together with major activities and duties. Based on this information, each occupation was coded according to the O^*^NET standard occupational classification (http://www.onetonline.org) by two independent raters. O^*^NET is the official occupational classification system of the U.S. Department of Labor, which codes occupations in a hierarchical structure and includes additional information on the skills and abilities required for the execution of each occupation.

AgeCoDe O^*^NET codes were derived as previously described (Forstmeier et al., 2012). In brief, two independent raters coded each AgeCoDe participant's longest-held occupation. Disagreements in coding were discussed with a third rater to reach consensus. The initial interrater agreement between the O^*^NET codes at the level of major groups (e.g., Life, Physical, and Social Science Occupations) was 86%. On the level of minor groups (e.g., Life Scientists and Physical Scientists), it was 74%. For specific occupations (e.g., Epidemiologist, Physicists, Chemists), the agreement was 66%.

In DELCODE, participants reported the main occupation (job title and main tasks) held in 5-year bands between the ages of 30 and 65 years, resulting in up to seven data points of occupation information per participant. Occupations were coded by four raters in total, with two raters independently coding half of the occupations of the first 394 participants and two raters coding half of the occupations of the remaining 683 participants. All ratings perceived as uncertain by any rater were discussed and reviewed at a consensus conference. To determine the interrater agreement, a random sample of 34 participants (238 occupational information data points) for the first 394 was drawn and coded by both raters. In addition, a random sample of 109 participants (677 occupational information data points) for the remaining 683 participants was drawn and coded by both raters. Initial agreement before the discussion of uncertain ratings was as follows: At the level of major groups in the O^*^NET system, the interrater agreement was 80% for the first 394 and 73% for the remaining 683 participants. At the level of minor groups, the agreement was 69 and 57%, respectively. At the level of specific occupations, it was 57 and 40%, respectively. The occupation most often listed across the 5-year bands was considered the longest-held occupation. If occupations were listed equally often, the maximum resulting OCRS associated with those occupations was used.

In both cohorts, housewives were coded as “personal and home care aides,” in line with previous research (Forstmeier et al., 2012). To build the OCRS in all cohorts, the level of cognition-related job activities of the longest-held occupation coded in O^*^NET (Supplementary Text 2) was summed in both cohorts, in line with the procedures described by Pool et al. (2016).

### Assessment covariates

In both cohorts, education was assessed as years of formal education. In AgeCoDe, APOE-ε4 was coded as either present (i.e., at least one APOE-ε4 allele) or absent.

### Assessment of cognitive outcomes

In AgeCoDe, global cognitive function was assessed using the MMSE (Folstein et al., 1983) at all assessments. In DELCODE, memory function was assessed using the memory factor score described by Wolfsgruber et al. (2020), which summarizes performance in the ADAS-Cog episodic memory tasks (Mohs et al., 1997), Free Cued and Selective Reminding Tests (Grober et al., 2009), Wechsler Memory Scale Logical Memory (Petermann and Lepach, 2012), CERAD figure recall (Thalmann et al., 2000), face–name association test (Polcher et al., 2017), and incidental learning of symbol number associations from the Symbol Digit Modality Test (Smith, 1982). As described in the introduction, memory was used as the primary outcome because of its strong link to AD and other neuropathologies (Wilson et al., 2019a). To assess the consistency of CR-related associations across cognitive domains, a global cognitive score (Wolfsgruber et al., 2020) was constructed as the average across factor scores of five cognitive domains (memory, executive function, working memory, visuospatial abilities, and language) and was used in a sensitivity analysis.

### Assessment of MRI markers in DELCODE

In DELCDOE, MRI markers were derived from images obtained at nine scanner sites (3T) according to procedures described previously (Jessen et al., 2018). Volumetric data were obtained automatically using FreeSurfer version 7 (cross-sectional pipeline) based on whole-brain T1-weighted (1 mm isotropic) and partial-volume T2-weighted images optimized for the medial temporal lobe (0.5 × 0.5 × 1.5 mm). A standard “recon-all-all” default pipeline was applied including intensity normalization, surface registration to Talairach space, skull stripping, and subcortical segmentation. Next, computation of the statistics of the segmented subcortical structures (Fischl et al., 2002), white matter segmentation, tessellation, and inflation of pial and white matter surfaces, followed by cortical parcellation and generation of the statistics of the parcellated cortical regions, were performed (Fischl et al., 2004). In addition, we performed automatic hippocampal subfield segmentation using high-resolution T2-weighted images to obtain the hippocampal volumes (Iglesias et al., 2015). These procedures were applied separately to MRI scans obtained at baseline and at the first follow-up. For the current analyses, the volumes of the left and right hippocampus were averaged. Temporal cortex thickness was obtained by averaging the thickness of all segmented regions belonging to the temporal cortex (bilateral).

### Assessment of CSF markers in DELCODE

CSF samples were collected by trained study assistants and processed, stored, and shipped to the central biorepository according to DZNE standardized operating procedures as previously described (Jessen et al., 2018). In brief, samples were aliquoted after collection, stored at −80°C in the DZNE biobank, and thawed once for ELISA measurement. Samples were assayed in technical duplicates, from which the mean and coefficient of variance (CV, percent of standard deviation divided by mean) of the duplicates were calculated. Samples with CV larger than 20% were repeated in measurement. On each ELISA plate, an eight-point calibrator curve, 39 samples, and one pooled and aliquoted internal reference CSF sample were measured. As DELCODE continuously and longitudinally collects samples, data were acquired throughout multiple ELISA plates and batches. The internal reference was used to control for the inter-run performance of the assay. Aβ42 and Aβ40 were quantified using the V-PLEX Aβ Peptide Panel 1 (6E10) Kit (K15200E), total Tau (tTau) was measured using V-PLEX Human Total Tau Kit (K151LAE) (Mesoscale Diagnostics LLC, Rockville, USA), and phospho-tau-181 (pTau181) was assessed using and INNOTEST PHOSPHO-TAU(181P) (81581; Fujirebio Germany GmbH, <city>Hannover</city>, Germany) assay according to vendor specifications. We used the ratio of Aβ42 to Aβ40 (Aβ42/40 ratio) to index amyloid pathology and ptau181 to index tau pathology in our study.

### Statistical analysis

All analyses were performed using R version 3.4.3. The significance threshold was set at α = 0.05.

#### Analysis of the association of the OCRS with CSF and MRI markers and cognition in DELCODE

Herein, the interaction of OCRS and AD biomarkers with cross-sectional memory function was assessed. Robust regression analyses were used, as implemented in the R package robustbase (Koller and Stahel, 2017; Maechler et al., 2018). Robust regression analyses were used to reduce the impact of extreme biomarker values observed in the DELCODE data. We modeled the interaction of the OCRS and either Aβ42/40 ratio, pTau181 (i.e., markers of AD pathology), hippocampal volume or temporal cortex thickness, and CSF total tau or total gray matter volume (i.e., global markers of neuronal loss) on memory function in separate models. In sensitivity analyses, the global cognitive score was used as the outcome.

In addition, the association of OCRS with all the aforementioned markers was assessed. Furthermore, the interaction of the OCRS and age with regard to CSF and MRI markers was estimated. CSF markers were log-transformed prior to analysis to approximate normal distribution. The association of OCRS with the estimated intracranial volume was also examined.

Furthermore, we tested the association of OCRS with longitudinal changes in all CSF and MRI markers using linear mixed models. Herein, we included only individuals with more than one marker assessment (CSF: *N* = 189; MRI: *N* = 606). Solely one follow-up assessment after 1 year was available for MRI markers (follow-up range: 0.77–1.58 years). Annual CSF assessments were repeated once in 146 individuals, twice in 40 individuals, and thrice in three individuals (follow-up range: 0.96–5.05 years). The lme4 package (Bates et al., 2015) was used to analyze the changes in CSF and MRI markers. Models with random intercepts were fitted using maximum likelihood estimation to account for repeated observations taken from the same individuals. Random slopes were not included because of the limited number of repeated observations. In the first step, the main effect of OCRS across all longitudinal observations was tested by including OCRS as a predictor in the mixed model. In the second step, we tested whether OCRS was associated with changes in markers from the baseline by including an interaction between OCRS and time from baseline to the model. Significance was assessed using the likelihood ratio test.

All analyses were controlled for age, sex, years of education, whether participants were already retired and the interaction of the variables with time. Retirement was included as a covariate because previous research has shown that retirement can affect cognition (Celidoni et al., 2017) and could relate to certain job characteristics. In the case of analyses of the MRI markers, we also controlled for intracranial volume at baseline and at the scanner site. Continuous predictors were z-standardized based on the total sample to facilitate the interpretation and comparability of the estimates. Patients with DAT were excluded from the sensitivity analysis.

In an exploratory analysis, the link of the OCRS with change in cognition was assessed (Supplementary Text 3).

#### Analysis of cognitive decline in AgeCoDe

To replicate the analyses of Pool et al. we analyzed the association of OCRS with cognitive decline in AgeCoDe. We used single-class univariate latent process mixed models, as implemented in the R package lcmm (Proust-Lima et al., 2011, 2017). Latent process mixed models estimate a latent process that represents the true level and change in a cognitive outcome and relate this latent process to observed data using a parameterized link function. This link function accounts for unequal interval scaling of cognitive outcomes, a common methodological limitation that is not considered by traditional statistical methods (Proust-Lima et al., 2011). To model the MMSE in AgeCoDe, different parametrized link functions (linear, quadratic I-splines with knots placed either equidistant across the outcome range or at percentiles of the distribution and beta cumulative distribution link function) were compared based on the Bayesian information criterion (BIC). In addition, the fixed effects of linear and quadratic time from baseline and the respective random effects were included and compared based on the BIC.

To assess the association of OCRS with cognitive decline, OCRS and its interactions with polynomials of time from baseline were modeled as fixed effects. Analyses were controlled for fixed effects of age, sex, years of education, and presence of at least one APOE-ε4 allele, as well as their interactions with polynomials of time (e.g., time^*^age, time^∧^2^*^age). To assess the interaction of OCRS with the APOE-ε4 allele, the three-way interaction of OCRS, APOE-ε4, and the respective polynomials of time were included in the fixed effects.

#### Analysis of cognitive decline prior to DAT onset in AgeCoDe

To assess whether the OCRS modulates cognitive change prior to dementia onset, generalized additive mixed models (Wood, 2004, 2011) (GAMM) were used as implemented in the R package mgcv (Wood, 2011). GAMM is a statistical method that allows the flexible consideration of non-linear associations between variables in longitudinal data. To this end, the link between a predictor and longitudinally measured outcome is modeled as a smooth function that can represent a very wide range of non-linear functional forms without the need for a priori assumptions about the shape of the functional form. Smooth functions can be modeled using different statistical techniques, such as (e.g., cubic regression or thin-plate regression) splines (Wood, 2003). Since the trajectory of cognition aligned to dementia onset can be expected to follow a highly non-linear shape (Amieva et al., 2014), GAMM offers a useful approach to model these data.

In this study, a total of 474 participants developing DAT during the follow-up of AgeCoDe and with information on OCRS were included, and their cognitive decline was assessed using the MMSE. Since GAMM does not allow for the inclusion of the beta cumulative distribution link function to account for non-equal interval scaling, the normalized version of the MMSE was used (Philipps et al., 2014), which accounts for the methodological problem based on an established normalizing transformation. First, to exclude the influence of extreme follow-up times in a few individuals on the model results, observations made in only 5% of the participants (i.e., >12.38 years before DAT onset and >3.31 years after DAT onset) were excluded.

Time to dementia onset was modeled using cubic regression splines, and the random slope of time relative to DAT onset and a random intercept were modeled to account for repeated measurements taken from the same individuals. In the next step, we included age, years of education, and OCRS as covariates and modeled their main effects on cognition using cubic regression splines. In addition, a fixed effect for sex and a time-varying indicator indexing the first MMSE assessment were included. The indicator of the first MMSE assessment was used to account for the practice effects which affect all measurements of the MMSE except for the first assessment. This was necessary because the first assessment of the MMSE (performed at the baseline of the study) was performed with different temporal lags relative to dementia onset (because individuals showed dementia conversion at different time points during follow-up). For example, for an individual converting at follow-up one, the assessment prior to dementia onset would be unaffected by the practice effect. In contrast, for an individual converting at follow-up 2, the assessment prior to dementia onset would be affected by the practice effect. This individual-specific influence needs to be considered to describe the natural trajectories of cognition independent of practice effects.

To assess the association of covariates with changes in cognition, tensor product interactions (Wood, 2006) (based on cubic regression splines) were fitted between the time to dementia onset and each continuous covariate. In addition, a smooth-factor interaction between time and sex (since sex is a categorical covariate) was included.

All analyses were fitted using maximum likelihood. Analyses were repeated using thin-plate regression splines (Wood, 2003), using all available observations and increasing the number of basis dimensions.

## Results

The descriptive statistics for the AgeCoDe and DELCODE participants in each analysis are presented in Table 1. Descriptive statistics for DELCODE stratified by diagnosis are provided in Supplementary Table 1.

**Table 1:** Descriptive statistics.

	**AgeCoDe**				**DELCODE**					
	**APOE sample**		**DAT converter sample**		**Whole cohort**		**MRI sample**		**CSF sample**	
	**Mean/*N***	**SD/%**	**Mean/*N***	**SD/%**	**Mean/*N***	**SD/%**	**Mean/*N***	**SD/%**	**Mean/*N***	**SD/%**
Age at baseline (Mean/SD)	79.37	3.45	80.33	3.49	71.27	6.17	71.11	6.12	71.13	6.04
Female sex (*N*/%)	1,553	65.6%	351	74.1%	441	50.5%	406	51.1%	226	48.6%
Years of education (Mean/SD)	12.05	2.28	11.89	2.25	14.49	3.00	14.51	3.01	14.35	2.91
MMSE at baseline (Mean/SD)	27.61	1.91	27.06	2.06	28.45	2.35	28.53	2.30	28.22	2.45
OCRS (Mean/SD)	3.17	0.85	3.05	0.82	3.94	0.82	3.95	0.82	3.94	0.84
Retired (*N*/%)	–	–	–	–	736	84.3%	670	84.4%	388	83.4%
APOE-ε4 carrier (*N*/%)	503	21.2%	135	28.5%	303	35.0%	265	33.7%	174	37.6%
**Biomarker of pathology at baseline**										
Aβ42/40 (Mean/SD)	–	–	–	–	0.08	0.03	0.09	0.03	0.08	0.03
pTau181 (Mean/SD)	–	–	–	–	61.14	31.98	60.17	31.83	61.14	31.98
tTau (Mean/SD)	–	–	–	–	451.51	269.21	448.87	267.96	451.51	269.21
HCvol (Mean/SD)	–	–	–	–	2,972.85	413.09	2,972.85	413.09	2,954.68	443.30
**Diagnostic groups**										
Controls (*N*/%)	–	–	–	–	201	23.0%	195	24.6%	82	17.6%
SCD (*N*/%)	–	–	–	–	364	41.7%	327	41.2%	188	40.4%
aMCI (*N*/%)	–	–	–	–	150	17.2%	129	16.2%	97	20.8%
DAT (*N*/%)	–	–	–	–	84	9.6%	75	9.4%	53	11.4%
DAT relatives (*N*/%)	–	–	–	–	74	8.5%	68	8.6%	45	9.7%
**Follow-up**										
Observation time (in years)	6.03	4.35	8.06	3.52	2.81	1.72	1.04^a^	0.08^a^	2.61^b^	0.91^b^

a Follow-up time specifically for MRI measures;

b follow-up time specifically for CSF measures. Aβ42/40, CSF Aβ42/Aβ42 ratio; aMCI, amnestic cognitive impairment; APOE, apolipoprotein E; CSF, cerebrospinal fluid; DAT, dementia of the Alzheimer's type; HCvol, average of left and right hippocampal volumes; N, sample size; OCRS, occupational cognitive requirement score; pTau181, CSF phospho-tau-181; SCD, subjective cognitive decline; SD, standard deviation; tTau, CSF total Tau.

### Association of the OCRS with MRI and CSF biomarkers and cross-sectional cognition in DELCODE

**Figure 3:**
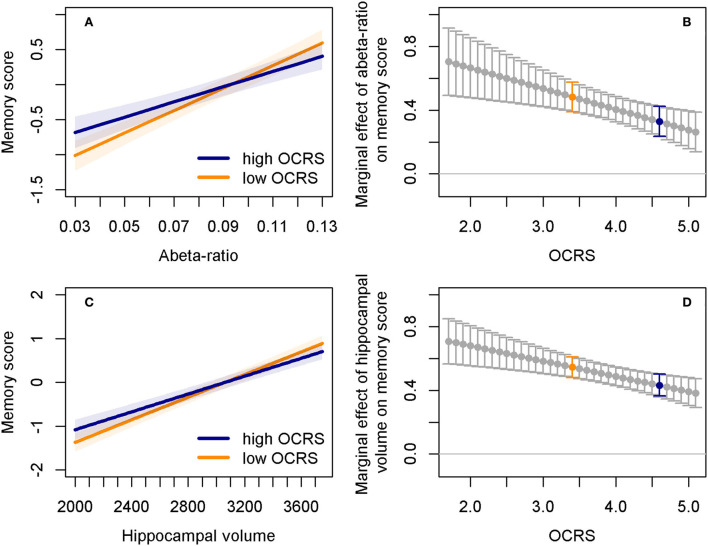
Interaction effects of OCRS with Aβ42/40 ratio and hippocampal volume regarding cross-sectional memory function. **(A)** Predicted memory factor scores depending on Aβ42/40 ratio in individuals with either low (25th percentile, orange line) or high (75th percentile, blue line) OCRS levels. Shaded areas indicate 95% confidence intervals. **(B)** Marginal effects of the Aβ42/40 ratio depending on OCRS levels. Bars indicate 95% confidence intervals. Marginal effects indicate the change in the memory factor when the Aβ42/40 ratio increases by one standard deviation. It is computed as the sum of the coefficients of the Aβ42/40 ratio and the Aβ42/40 ratio*OCRS interaction term. Blue dots and bars correspond to the predicted trajectory for individuals with high OCRS (blue line) in plot **(A)**. Orange dots and bars correspond to the predicted trajectory for individuals with low OCRS (orange line) in plot **(A)**. Marginal effects indicate that effects of Aβ42/40 ratio on memory function are stronger at lower levels of the OCRS. **(C)** Predicted memory factor scores depending on the averaged left and right hippocampal volume in individuals with either low (25th percentile, orange line) or high (75th percentile, blue line) OCRS levels. Shaded areas indicate 95% confidence intervals. **(D)** Marginal effects of the hippocampal volume depending on OCRS levels. Bars indicate 95% confidence intervals. Interpretation analogous to **(B)**, that is, marginal effects indicate that the effects of hippocampal volume on memory function are stronger at lower levels of the OCRS. Abeta ratio, cerebrospinal fluid Aβ42/Aβ42 ratio; OCRS, occupational cognitive requirement score.

First, we explored whether OCRS mainly acts through CR, BM, or BR. Herein, we turned to the DELCODE study, where direct measurements of pathology are available in participants across a broad spectrum of clinical impairments and AD risks. Interaction analyses using robust regression models (Supplementary Table 2) showed a reduced association of the CSF Aβ42/40 ratio (b = −0.11, SE = 0.04, *p* = 0.003, Figures 3A,B) with memory function in individuals with higher as compared to lower OCRS. No modification in the association of CSF pTau181 with memory function by OCRS was found. In contrast, high OCRS was associated with a reduced association of hippocampal volume (Est = −0.08, SE = 0.03, *p* = 0.001, Figures 3C,D) and temporal cortex thickness (Est = −0.09, SE = 0.03, *p* = 0.0007) with memory function. There was a trend-level interaction effect of OCRS and total gray matter volume (Est = −0.05, SE = 0.03, *p* = 0.061), but no interaction between OCRS and tTau. The results on Aβ42/40 ratio, hippocampal volume, and temporal cortex thickness were significant after Bonferroni correction for multiple testing. Repeating analyses with global cognition as the outcome (Supplementary Table 3) replicated results on memory function, but additionally showed an interaction between OCRS and total gray matter volume (Est = −0.05, SE = 0.02, *p* = 0.039). All analyses were adjusted for age, sex, and years of education. The results did not change substantially when patients with DAT were excluded, although the interaction effects of the OCRS with MRI markers were reduced and only significant at the trend level (Supplementary Tables 2, 3). Further excluding MCI patients from the sample resulted in no significant interaction effects in our sample, probably due to variance restriction and selection bias (Supplementary Table 4; see Section Moderating role of OCRS in the link between directly measured pathology and cross-sectional cognitive function for discussion).

Furthermore, we found that the OCRS itself was not related to the cross-sectional levels of Aβ42/40 ratio and pTau (Supplementary Table 5). There was a cross-sectional positive association of the OCRS with hippocampal volume (Est = 37.16, SE = 14.37, *p* = 0.009), temporal cortex thickness (Est = 0.01, SE = 0.01, *p* = 0.048), and total gray matter volume (Est = 5,731.9, SE = 1,490, *p* = 0.0001), but no association with tTau (Supplementary Table 5). However, the association of OCRS with MRI markers did not depend on age, as indicated by the absence of OCRS × age interactions (Supplementary Table 5).

In line with these cross-sectional results, longitudinal data analyses (CSF: *N* = 189, number of observations = 424; MRI: *N* = 606, number of observations = 1,212; Supplementary Table 6) showed an association between the OCRS and general levels of hippocampal volume (Est = 37.77, SE = 16.43, *p* = 0.020) and general levels of total gray matter volume (Est = 5,164.0, SE = 1,645.1, *p* = 0.001) across all longitudinal assessments. We found no association between Aβ42/40 ratio, pTau, temporal cortex thickness, or tTau (Supplementary Table 6). In addition, there was no association between OCRS and longitudinal change from baseline in any pathologic marker (Supplementary Table 6). The results were similar when individuals with DAT were excluded (Supplementary Table 6). When excluding MCI and DAT patients from the sample, only the cross-sectional association of the OCRS with total gray matter volume remained (Supplementary Tables 7, 8). Controlling for follow-up time did not change the results substantially (Supplementary Table 8).

OCRS did not predict the estimated intracranial volume (whole sample: Est = 9,026, SE = 7,063, *p* = 0.202; excluding DAT cases: Est = 13,661, SE = 7,453, *p* = 0.067).

Exploratory analyses of longitudinal cognitive change (Supplementary Text 3) revealed a just significant interaction of OCRS and temporal cortex thickness regarding cognitive decline in the analysis excluding patients with MCI and DAT [Chi^2^_(2)_ = 6.042, *p* = 0.049; Supplementary Tables 9–11]. This association did not survive correction for multiple testing. No other significant associations with cognitive change were found.

### Longitudinal cognitive decline in AgeCoDe

**Figure 4:**
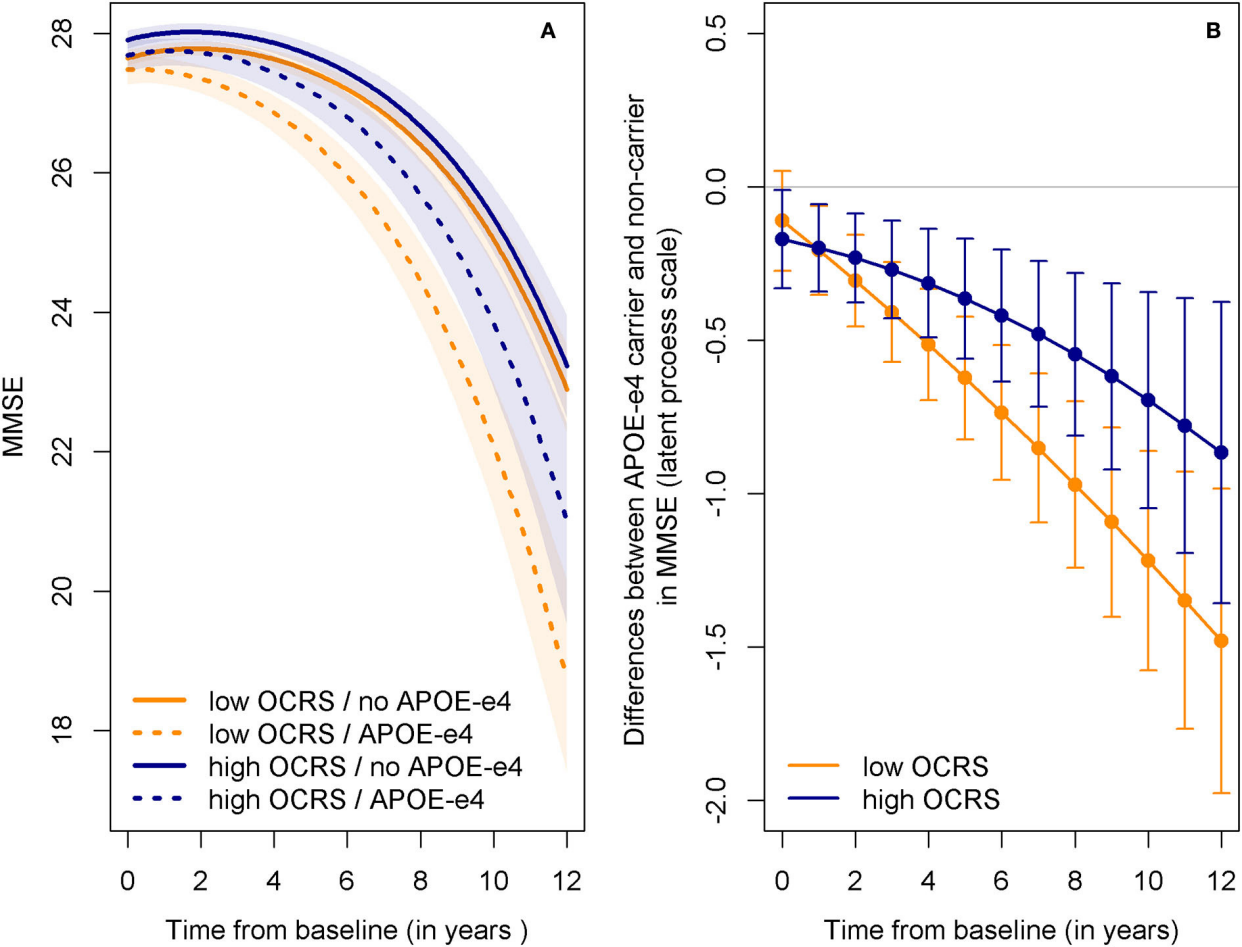
Predicted trajectories of cognitive decline in AgeCoDe depending on OCRS and APOE-ε4. **(A)** Predicted trajectories in MMSE for APOE-ε4 carrier and non-carrier with either low (25th percentile) or high (75th percentile) OCRS levels. Shaded areas indicate 95% confidence intervals. While APOE-ε4 is generally associated with a stronger cognitive decline (steeper slope for dotted compared to straight lines), this difference is larger in individuals with low OCRS (orange lines) compared to high OCRS (blue lines). **(B)** Differences in MMSE between APOE-ε carrier and non-carrier at different time points for individuals with either low (25th percentile) or high (75th percentile) OCRS levels. Bars indicate 95% confidence intervals. Differences are presented on the scale of the latent variable in the latent process mixed models (Proust-Lima et al., 2011), not on the scale of the observed variable (i.e., the MMSE). Differences are generally lower (i.e., closer to zero) for individuals with high (blue line) as compared to low (orange line) OCRS values. APOE-e4, apolipoprotein E ε4 allele; MMSE, Mini-Mental-State-Examination; OCRS, occupational cognitive requirement score.

Next, we studied the association of the OCRS with cognitive decline in the MMSE in AgeCoDe to replicate the results of Pool et al. (2016). The lowest BIC suggested that latent process mixed models of MMSE trajectories were best represented by models including a random intercept and random slope of time and time squared, as well as a beta cumulative distribution link function to adjust for non-equal interval scaling (Proust-Lima et al., 2011). Models adjusted for age, sex, educational level, and carrying the APOE-ε4 allele showed no association of OCRS with the speed of cognitive decline in all AgeCoDe participants with information on OCRS [Chi^2^_(2)_ = 0.314, *p* = 0.855, Supplementary Table 12]. However, individuals with high OCRS and carrying the APOE-ε4 allele had a significantly weaker association with cognitive decline than individuals with low OCRS [Chi^2^_(2)_ = 6.931, *p* = 0.031, Supplementary Table 12], as shown by predicted MMSE trajectories (Figure 4A). In addition, we observed a reduced difference between APOE-ε4 allele carriers and non-carriers in MMSE decline at high levels of OCRS (Figure 4B). Notably, higher OCRS was associated with higher baseline levels of cognition (Supplementary Table 12).

### Longitudinal cognitive decline prior to DAT onset in AgeCoDe

**Figure 5:**
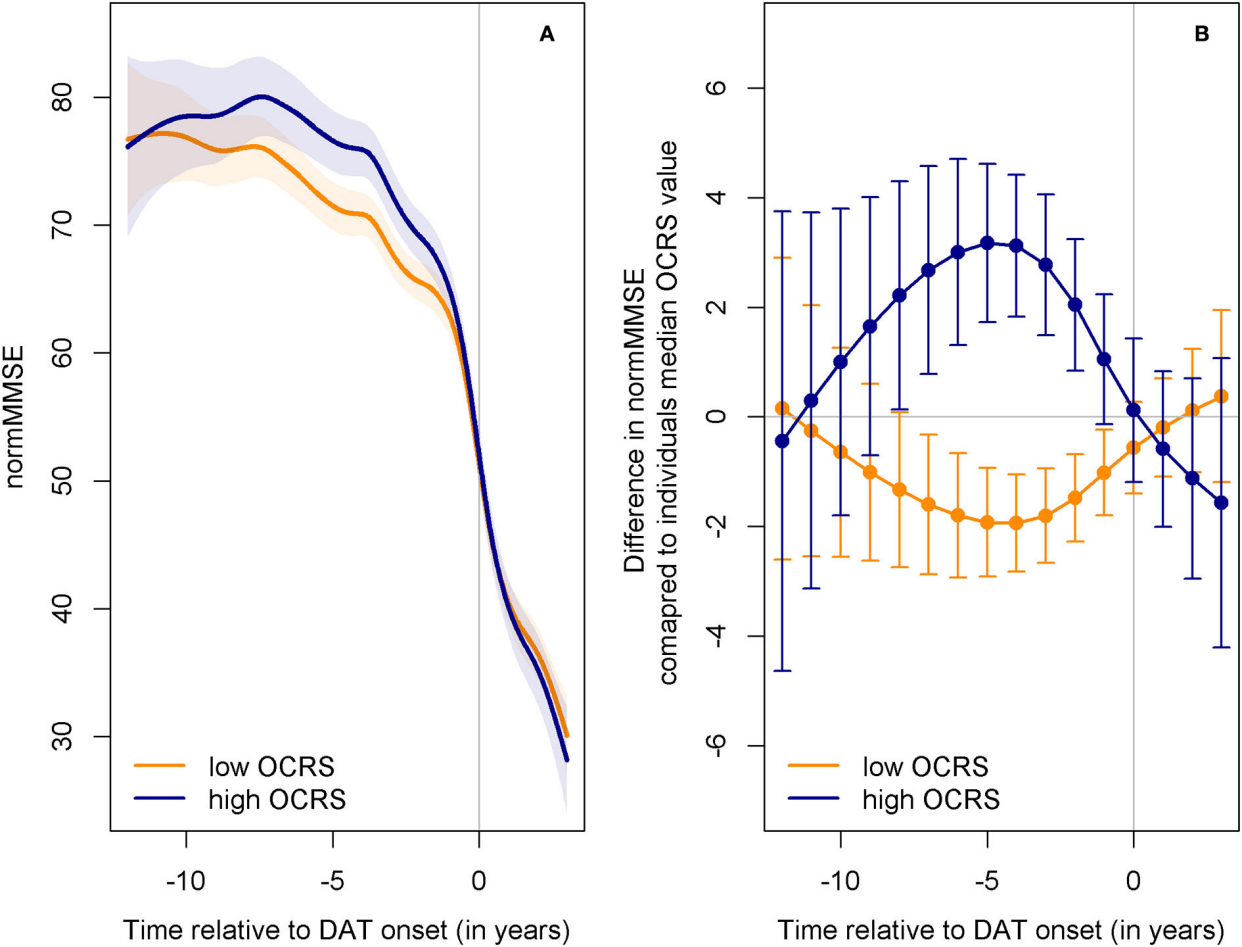
Predicted trajectories of cognitive decline in AgeCoDe before DAT onset depending on OCRS. **(A)** Predicted trajectories in the normalized MMSE (normMMSE) (Philipps et al., 2014) relative to the onset of DAT for individuals with either low (25th percentile) or high (75th percentile) OCRS levels. The normalized MMSE has a range of 0 to 100. Shaded areas indicate 95% confidence intervals. Individuals with high OCRS (blue line) show stable cognitive function for a longer period than individuals with low OCRS (orange line). However, they decline stronger after the onset of deterioration. Both groups show equal levels of performance at dementia onset. Afterward, high OCRS is associated with slightly lower levels of cognitive function. **(B)** Predicted differences of individuals with high (75th percentile) or low OCRS (25th percentile) compared to individuals with median OCRS values at different time points relative to DAT onset. Bars indicate 95% confidence intervals. Differences are computed from the sum of OCRS smooth terms [ti(OCRS) in the mgcv package] and OCRS and time tensor product interaction terms [ti(time,OCRS) in the mgcv package]. While high OCRS (blue line) shows an increasingly protective association with cognitive function until ~5 years before dementia onset, this association diminishes and predicted cognition is even lower than in individuals with low OCRS (orange line) after DAT onset. DAT, dementia of the Alzheimer's type; normMMSE, normalized Mini-Mental-State-Examination; OCRS, occupational cognitive requirement score.

Finally, MMSE trajectories aligned to dementia onset in AgeCoDe participants who progressed to DAT were modeled to complement previous empirical tests of conceptual predictions using the large prospective AgeCoDe study. The GAMM showed that the OCRS significantly modified the shape of the MMSE trajectory (*F* = 3.26, edf = 6.61, *p* = 0.004, Supplementary Table 13, Figure 5). Plots illustrating the non-linear association of the OCRS prior to DAT onset over the entire range of the variables are shown in Supplementary Figure 2. As can be seen from these figures, a high OCRS is associated with stable or even slightly increasing cognitive performance until ~5 years before DAT diagnosis. During the same period, a low OCRS was already associated with a slight cognitive decline. Subsequently, high OCRS is associated with a stronger cognitive decline, whereas low OCRS is associated with a more gradual, lower cognitive decline.

## Discussion

**Figure 6:**
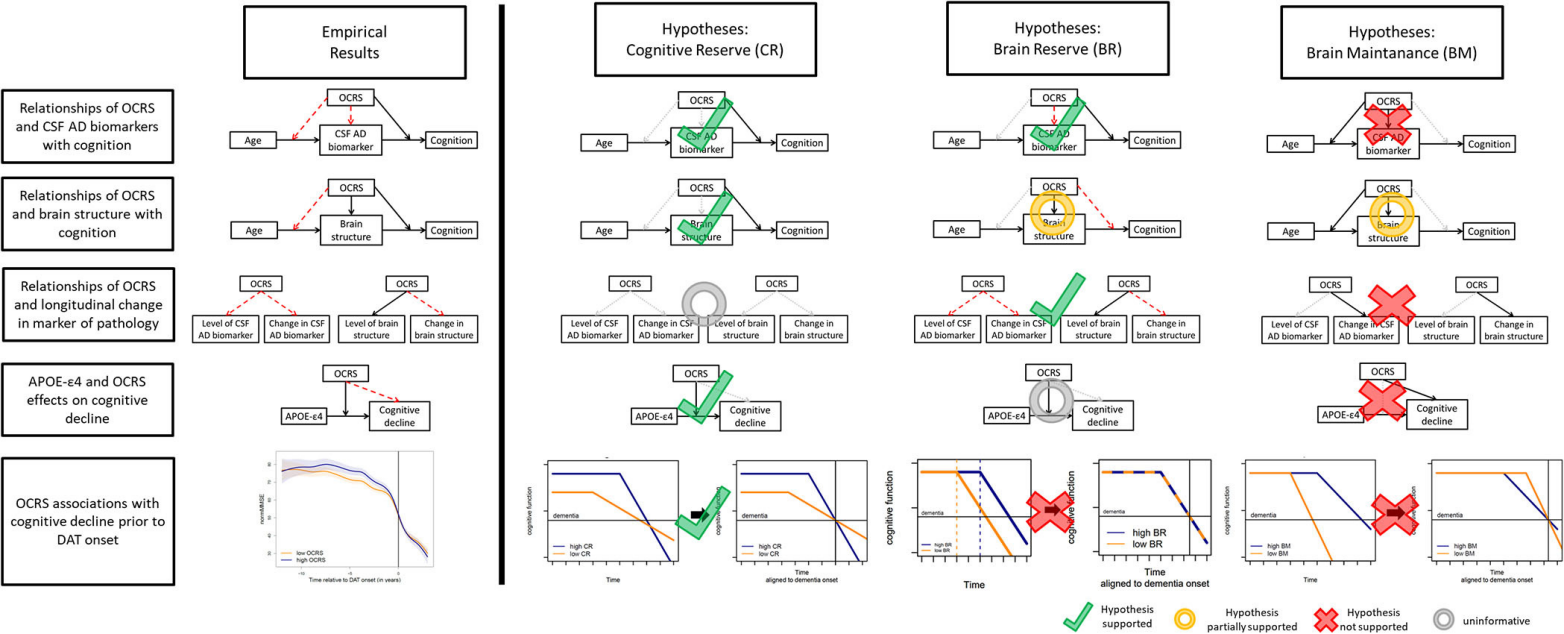
Graphical illustrations of hypothesis test results for CR, BM, and BR. In the graphical illustrations of hypotheses **(right side)**, black arrows linking boxes indicate expected association, red dashed arrows indicate associations inconsistent with the predictions by the respective resilience concept, and gray dotted arrows indicate no expectation regarding association by the respective resilience concept. Arrows pointing toward other arrows indicate an expected statistical interaction effect. In the bottom row, plots on the left illustrate the typical development of cognition over time. Bold arrows indicate the alignment of time to the onset of dementia (horizontal black line) that is a shift along the x-axis. In the graphical illustrations of empirical results **(left side)**, black arrows indicate supported associations while red arrows indicate non-significant associations not supported by our data. OCRS, occupational cognitive requirement score; APOE-ε4, apolipoprotein E ε4 allele; CSF, cerebrospinal fluid; CR, cognitive reserve; BM, brain maintenance; BR, brain reserve.

This study aimed to examine whether work-related cognitive activities in midlife, as captured with the OCRS (Pool et al., 2016), protect against cognitive decline in old age, and whether such a protective association would be based on CR, BR, or BM (see Figure 6 for a graphical summary of the results).

### Moderating role of OCRS in the link between directly measured pathology and cross-sectional cognitive function

We found a reduced association of Aβ42/40 ratio with cross-sectional memory and global cognitive function in participants with higher OCRS in DELCODE, supporting a link between the OCRS and CR or BR, since a reduction in the impact of AD pathology on cognitive function is expected by these concepts. In contrast, the absence of any longitudinal association of the OCRS itself with any AD CSF level does not support a relationship with BM, as the rate of accumulation of pathology should be lower in individuals with high BM. The absence of cross-sectional differences (especially at older ages) does again not support a link of the OCRS to BM.

When focusing on measures of brain structure and neural loss, we again found support for a link between OCRS and CR mechanisms, as high OCRS was associated with a reduced cross-sectional association of hippocampal volumes and temporal cortex thickness with memory and global cognitive function. In contrast, the BR theory does not expect this reduced association because the protective effects of BR should be derived from the brain structure itself and should therefore be lost (or at least substantially diminished) once the brain structure itself is reduced. In addition, there was no association between OCRS and intracranial volume, a common proxy for BR, which does not support the connection of OCRS to this concept. However, we observed a cross-sectional association of the OCRS with hippocampal volume, temporal cortex thickness, and total gray matter volume, as expected by BR and BM theory. This finding is consistent with previous research linking occupational activity with brain structure (Suo et al., 2012; Kaup et al., 2018; Habeck et al., 2019; Rodriguez et al., 2021a). Notably, but contrary to predictions based on BM theory (Nyberg et al., 2012; Steffener et al., 2014), the cross-sectional association of OCRS with brain structural measures was not stronger in older individuals and was not detectable with regard to longitudinal changes in MRI markers. However, the short follow-up in the currently available DELCODE data limited our ability to detect longitudinal changes in the markers of pathology, and survival bias may have affected the interaction between OCRS and age regarding the markers. Previous studies examining the link of other proposed proxy measures of CR with longitudinal change in markers of pathology have not found clear evidence for a consistent association as shown in a recent review by Soldan et al. (2020). Our results on the OCRS are consistent with these findings, but more research in larger studies with longer follow-up on pathologic markers is required for confirmation.

In summary, these analyses support the predictions made by CR theory, but only partially support the expectations derived from BR and BM theory.

Of note, interactions of the OCRS with markers of pathology regarding cross-sectional cognitive function supporting a link to CR were not present when excluding MCI patients. However, excluding MCI patients may have artificially attenuated the effect of CR on the interplay of cognition and pathology. Both MCI and SCD patients (the largest group in the remaining sample) were recruited for memory clinics and delineated based on their cognitive performance. Thus, at the same level of pathology, individuals with high CR are more likely to receive an SCD diagnosis (due to better compensation of pathology), while individuals with low CR are more likely to receive an MCI diagnosis (due to less compensation). Excluding individuals with MCI, therefore, depletes the sample from individuals with low CR or higher levels of pathology thereby counteracting the ability for detecting interaction effects consistent with CR in the DELCODE sample. In line with this, MCI patients descriptively showed lower levels of OCRS if they also showed higher levels of pathology as compared to SCD patients. Nevertheless, further research on cognitively normal individuals recruited from the general population is needed to test whether a link of the OCRS with CR can be shown in cognitively normal individuals.

### OCRS and cognitive decline in older individuals

We found that higher OCRS is not generally related to slower cognitive decline in individuals aged 75 years or above from the AgeCoDe cohort; instead, it is associated with a reduction in the association of carrying an APOE-ε4 allele with cognitive decline. Notably, it was previously shown in AgeCoDe that specific cognitive demands are especially important in mitigating the relationship between APOE-ε4 and cognitive decline (Rodriguez et al., 2021b). In the current study, we provide novel evidence that the OCRS is a reliable and easily implementable global measure for assessing work-related cognitive requirements that are associated with preserved cognitive function in individuals at a genetically elevated risk for pathology. As shown in our study, the information provided by the OCRS extends beyond the information included in the educational level and should therefore complement assessments of protective cognitive activities. Our results differ from those of Pool et al. (2016), who found a general protective association with cognitive decline across all participants, but no significant interaction of the OCRS with APOE-ε4 (*p* = 0.11). Of note, previous research examining the interaction between education, a prominent additional proxy measure of CR, and APOE genotypes regarding cognitive decline also revealed inconsistent results. Two studies (Seeman et al., 2005; Van Gerven et al., 2012) found the strongest cognitive decline in highly education APOE-ε4 carrier, while other studies showed no significant statistical interaction (Kalmijn et al., 1997) or a decreasing association of APOE-ε4 with cognitive decline as education increases (Shadlen et al., 2005). Only the latter finding is consistent with our results on the interaction of APOE-ε4 with the OCRS regarding cognitive decline in AgeCoDe. Potential explanations for inconsistencies should be investigated in future research. We will discuss one possible reason below in light of our results on the link between the OCRS and CR, BM, and BR (Section Implications).

Regarding the distinction between these resilience concepts, only suggestive indications can be derived in the absence of a direct assessment of pathology. Considering this, the pattern of our results on OCRS and APOE-ε4 statistical interaction effects on cognitive decline is most consistent with OCRS mainly acting through CR or BR since the OCRS mitigates the association between APOE-ε4 and cognitive decline. In contrast, the results are not consistent with the involvement of the OCRS in BM mechanisms, as an overall protective association with cognitive decline (as expected by BM theory) was not found.

### OCRS and trajectories of cognitive decline aligned to dementia onset

Similarly, analyses of cognitive trajectories aligned with the onset of DAT support a link between the OCRS and CR, as we observed the expected longer preservation of cognitive function with a stronger decline afterward in those with higher OCRS. In contrast, BM would have predicted an earlier onset and, afterward, slower rate of decline in those with high OCRS. BR would have predicted no difference between individuals in the trajectories depending on the OCRS. Interestingly, similar trajectories have been found for the association of education, a well-known CR proxy, with cognitive decline before dementia onset (Amieva et al., 2014), emphasizing the feasibility of this approach to gain insights into the link between lifestyle factors and CR and related concepts. In addition, the stronger decline after the onset of impairment found in our study is consistent with previous research on CR effects in MCI patients (Myung et al., 2017). Nevertheless, the results derived from this approach should be considered suggestive evidence and require further investigation in cohorts with a direct assessment of pathology. Notably, in our study, the results obtained using this approach were consistent with the findings from DELCODE, providing a direct assessment of pathology. It is, therefore, tempting to speculate that studying cognitive trajectories aligned with dementia onset could provide a new opportunity to generate hypotheses on the most likely resilience mechanism in cohorts lacking a direct assessment of pathology. Since this would allow more cohorts and researchers to study resilience, it could help examine the generalizability and disparities in these concepts. However, more studies are needed to check whether the results from this approach reliably correspond to those obtained from analyses using direct assessments of pathology. Of note, change point models could provide another highly useful methodological approach to examine the onset and rate of change in cognition relative to dementia onset (Karr et al., 2018). These models have previously been successfully used to examine the effect of CR proxies on cognitive trajectories (Hall et al., 2007, 2009; Wilson et al., 2019b).

### Implications

Our results suggest a stronger link between midlife cognitive activities, as indexed by the OCRS, and CR as compared to BM or BR. In line with these results, previous research on work-related cognitive activities in midlife has consistently shown a protective role in the risk of dementia and cognitive decline (Kröger et al., 2008; Smart et al., 2014; Pool et al., 2016; Then et al., 2017) beyond education. Similarly, higher levels of pathology (at the same level of cognitive function) have been observed for individuals with more complex and cognitively demanding occupations, as suggested by the CR theory (Stern et al., 1995; Garibotto et al., 2008; Boots et al., 2015). No statistical interaction analyses were performed in these studies.

Of note, established proxy measures of CR show interactions with markers of pathology regarding cross-sectional cognition which are similar to the OCRS. For instance, higher education is associated with a reduced effect of amyloid pathology (Joannette et al., 2020) and white matter hyperintensities (Dufouil et al., 2003; Zahodne et al., 2019) on memory function. Early life cognitive abilities, as another proxy of CR, have been shown to attenuate the association of hippocampal with memory function in midlife (Vuoksimaa et al., 2013). However, not all studies found such an association for education (Malek-Ahmadi et al., 2017) or other reserve proxies (Vemuri et al., 2011).

Taken together, these results point to the relevance of stimulating midlife cognitive activities for dementia prevention. Importantly, several studies suggested prevention measures (Livingston et al., 2020), and, in particular, pharmacological interventions target a reduction in age-related pathologies. Cognitive activities in midlife, in turn, seem to promote CR (i.e., resistance to those pathologies), thereby contributing to dementia prevention through a complementary mechanism. Therefore, promoting cognitive activities in midlife should be considered as a complementary approach to early dementia prevention measures.

Furthermore, if (as our results suggest) midlife cognitive activities truly act through CR, their beneficial effect will be most pronounced in individuals at high risk for developing pathologic brain changes or in old age, where pathologic changes are highly prevalent. Future research on factors promoting CR in midlife should focus on these groups when assessing the suspected protective effects.

Future research should focus on refining specific interventions and activities that promote cognitive function and potentially CR-related mechanisms in midlife, since there are currently limited data to recommend conducting any specific cognitive activity or training to reduce dementia risk (Butler et al., 2018). Given that the OCRS captures occupational cognitive activities, it is tempting to speculate that enriching work environments, for example by proving regular advanced training offers, might positively affect cognition and CR.

While our results support a link between OCRS and CR in old age and individuals at elevated risk for AD, they do not exclude the possibility that the protective role of OCRS may be additionally conveyed by mechanisms other than CR. The OCRS might affect cognitive function by more than one resilience mechanism. Importantly, interactions between resilience mechanisms and differential sensitivity of our analyses to the specific hypotheses derived for each concept may have hampered links to resilience concepts beyond CR. Other lifestyle factors have been proposed to act through more than one mechanism (Arenaza-Urquijo et al., 2015; Chételat, 2018). Previous research has proposed that lifestyle factors may predominately act *via* neuroprotection (i.e., BM) in younger individuals or in the early phase of pathology accumulation, but then mainly act through CR as more pathology develops (Arenaza-Urquijo et al., 2015; Chételat, 2018). Therefore, the OCRS may show a different pattern of associations in other age strata or in other target populations. Interestingly, this hypothesis might explain why we could not completely replicate the results of Pool and colleagues (Pool et al., 2016) as the age at baseline in their study was considerably lower than that of AgeCoDe (Pool et al.: 26% ≥75 years vs. AgeCoDe: 100%≥75 years). Consequently, the higher age at baseline might have increased the power to detect the interaction of OCRS with APOE-ε4 in our study and reduced the likelihood of detecting the association with cognitive decline in the whole cohort. Systematic examination of the effect of age on the protective impact of lifestyle factors may provide additional valuable insights into the potential mechanisms conveying their effects.

Furthermore, our results on cognitive trajectories before dementia onset may have implications for the assessment of associations of CR-related factors in longitudinal cognitive data derived from cohorts enriched for individuals at risk for dementia, such as the memory clinic-based DELCODE cohort. In our AgeCoDe analyses, we observed that OCRS, as a potential marker of CR, was initially associated with a slower rate of cognitive decline. However, closer to the onset of dementia, it is associated with a faster rate of decline. Since the time to dementia onset is unknown for most memory clinic patients, our observation of predementia trajectories implies that time-dependent associations of CR markers may counteract each other in the longitudinal data and could cancel out. Similarly, when analyzing the interaction of CR markers with markers of pathology regarding longitudinal cognition, our results on predementia trajectories would imply that the direction of the interaction between CR and pathology markers will change nonlinearly over time, which is very difficult to model. However, in cross-sectional data, the influence of the time dependency of the association will be less severe because individuals with high CR should still show better cognition compared to individuals with lower CR close to dementia onset, despite a faster rate of decline. In line with this, we did not observe differential associations between changes in cognitive function and pathologic markers depending on the OCRS (modeled linearly) in the memory clinic-based DELCODE cohort (Supplementary Tables 9–11). Available sample size and limited follow-up on biomarker assessments precluded a more fine-grained assessment of the longitudinal, possible linear interplay of the OCRS with pathology and cognition. Future research needs to assess whether the effects of the OCRS described in DELCODE are only restricted to processes acting early during the development of pathology (and might have manifested as baseline cognitive differences in DELCODE) or whether these processes are also important for later stages of pathologic changes.

### Strengths and limitations

A strength of our study is the use of two independent cohorts that provide converging evidence from a large population-based study (AgeCoDe) and from a deeply phenotyped clinical cohort (DELCODE), with direct measures of certain pathologies. The hypotheses and operationalization generated for each of the research settings might be reused and adapted by future studies on other lifestyle factors and reserve proxies. Importantly, the statistical interaction effect of reserve proxies with markers of pathology on cognitive function has been proposed as the gold-standard test for CR (Stern et al., 2020) and has not been applied in most studies evaluating the link between midlife cognitive activities and CR. Of note, Udeh-Momoh et al. (2019) showed that a professional-level occupation reduces dementia risk associated with high levels of amyloid pathology and cortisol pointing toward a need to study the role of stress in the link between occupation and cognitive decline. Furthermore, our hypotheses derived for each resilience concept regarding trajectories of cognitive decline prior to the onset of dementia add a novel perspective. Such analyses may allow for future exploratory research in cohorts that lack a direct assessment of pathology. Moreover, replicating the results on the protective role of the OCRS, which was originally developed based on job characteristics in the USA, in two German samples, allowed us to demonstrate the generalizability of the results of the OCRS across societies and languages.

However, our study has some limitations. A direct assessment of pathology was missing in the AgeCoDe cohort, allowing only indirect and less precise tests of the links of the OCRS to BM and BR. Furthermore, our analyses of longitudinal changes in MRI markers in DELCODE were based on pre-processing procedures that considered different time points separately. Longitudinal MRI processing (Reuter et al., 2012), which is probably more sensitive to individual atrophy over time, would provide more certainty in definitely ruling out an association between OCRS and BM. Furthermore, the short follow-up duration in DELCODE has limited our sensitivity for the detection of age-related changes in CSF and MRI measures and might have hampered the detection of a link between OCRS and BM. In addition, sparse data on individuals with low levels of pathology and high OCRS in DELOCDE have limited our ability to reliably assess the association of OCRS with cognition in individuals without pathology. In addition, the cognitive task used in the population-based cohort might have been too insensitive to detect the effects of the OCRS conveyed by BM, that is, reduced accumulation of age-related pathology.

Moreover, we cannot show that the associations described are exclusively related to midlife cognitive activities. While the OCRS was developed to measure these activities, other (unmeasured) factors, such as socioeconomic status or general health and healthcare access, may have contributed to the association of the OCRS with CR in our cohort. Furthermore, from cross-sectional analyses, the directions of the effects underlying the observed associations are uncertain. For instance, individuals with more efficient brains may have worked in jobs involving more cognitive activities. However, regardless of the particular mechanism, high OCRS may serve as a marker for high CR. This might be helpful in defining effective and personalized prevention measures for individuals in future. Previous research has shown that cognitive and physical activity (Andel et al., 2015, 2016) can be more strongly associated with cognition and dementia risk reduction in individuals with less complex occupations.

Furthermore, the OCRS is based on general job characteristics from the O^*^NET database, which does not capture the individual work experiences and subjective perceptions of each participant. This may reduce the precision of the assessment of work-related occupational demands.

## Conclusion

Our study demonstrates that high OCRS is associated with a reduced association of APOE-ε4 and AD biomarkers with cognitive decline and memory function, respectively. Furthermore, high OCRS is associated with a slightly later onset and steeper slope of cognitive decline prior to dementia. These results suggest that OCRS is a valid indicator of CR, a resilience mechanism that mitigates the effects of emerging pathology on cognitive functioning.

## Data availability statement

The datasets presented in this article are not readily available because legal and privacy issues as well as ethical considerations do not allow making the data used in this study publicly available. However, access to the data can be granted by the data handeling center and the steering commitees of the AgeCoDe or DELCODE study for research purposes upon reasonable request. Requests to access the datasets should be directed to Luca.Kleineidam@ukbonn.de.

## Ethics statement

The studies involving human participants were reviewed and approved by the Ethics Commission of the Medical Association Hamburg, Ethics Committee of the Medical Faculty of the Rheinische Friedrich-Wilhelms-University of Bonn, Medical Ethics Commission II of the Medical Faculty Mannheim/Heidelberg University, Ethics Committee at the Faculty of Medicine of the University of Leipzig, Ethical Committee of the Medical Faculty of the Heinrich-Heine-University Düsseldorf, Ethics Committee of the Faculty of Medicine of the Technical University of Munich (AgeCoDe study). The DELCODE study was approved by the Ethical Committee of the medical faculty of the University of Bonn and approved by all participating sites Ethical Committees [Ethical Committee of the Charité Berlin, Ethical Committee of the medical faculty of the University of Bonn, Ethical Committee of the Univeristy of Cologne, Ethical Committee of medical faculty of the University of Göttingen, Ethical Committee of the Univeristy of Magdeburg, Ethical Committee of the Univeristy of LMU (Ludwig-Maximilians-University) Munich, Ethical Committee of the Univeristy of Rostock, Ethical Committee of the Univeristy of Tübingen]. The patients/participants provided their written informed consent to participate in this study.

## Members of the AgeCoDe and AgeQualiDe study group

^*^Principal Investigators: Wolfgang Maier, Martin Scherer, and Steffi G. Riedel-Heller. Heinz-Harald Abholz, Christian Brettschneider, Cadja Bachmann, Horst Bickel, Wolfgang Blank, Hendrik van den Bussche, Sandra Eifflaender-Gorfer, Marion Eisele, Annette Ernst, Angela Fuchs, André Hajek, Kathrin Heser, Frank Jessen, Hanna Kaduszkiewicz, Teresa Kaufeler, Mirjam Köhler, Hans-Helmut König, Alexander Koppara, Diana Lubisch, Tobias Luck, Dagmar Lühmann, Melanie Luppa, Tina Mallon, Manfred Mayer, Edelgard Mösch, Michael Pentzek, Jana Prokein, Alfredo Ramirez, Susanne Röhr, Anna Schumacher, Janine Stein, Susanne Steinmann, Franziska Tebarth, Carolin van der Leeden, Michael Wagner, Klaus Weckbecker, Dagmar Weeg, Jochen Werle, Siegfried Weyerer, Birgitt Wiese, Steffen Wolfsgruber, and Thomas Zimmermann. ^*^Hendrik van den Bussche (2002–2011).

## Author contributions

LK conceptualized the study, conducted the statistical analyses, and drafted the manuscript. MW and SR-H contributed to the conceptualization and supervised the study. SWo and A-SW contributed to statistical analyses. A-SW, LZ, and SFo contributed to the acquisition, interpretation, and analysis of occupational data. LK, SWo, A-SW, LZ, SFo, SR, HBu, HK, BW, SWe, JWe, AF, MP, CBr, H-HK, DW, HBi, ML, FR, SFr, SE, CU, OP, ES, SA, AL, JP, KF, XK, ASc, CBa, BS, JWi, FM, WG, EI, MB, ED, KB, DJ, ME, B-SR, RP, IK, DG, ST, CL, MM, ASp, NR, FB, MH, AR, RY, MS, WM, FJ, SR-H, and MW substantially contributed to the acquisition and/or interpretation of the data. All authors reviewed and critically revised the manuscript for important intellectual content.
