# Supplementary material for: Midlife occupational cognitive requirements protect cognitive function in old age by increasing cognitive reserve
Source: Front Psychol. 2022 Dec 8;13:957308. doi: 10.3389/fpsyg.2022.957308 (PMC9773841; doi:10.3389/fpsyg.2022.957308)
Supplement: Supplementary file 1 [file Data_Sheet_1.pdf]

Supplementary file

Mid-life occupational cognitive requirements protect cognitive function in old age by increasing cognitive reserve

Luca Kleineidam[1,2], Steffen Wolfsgruber[2], Anne-Sophie Weyrauch[1], Linn E. Zulka [1,36], Simon Forstmeier[3], Sandra Roeske[2], Hendrik van den Bussche[4], Hanna Kaduszkiewicz[4,5], Birgitt Wiese[6], Siegfried Weyerer[7], Jochen Werle[7], Angela Fuchs[8], Michael Pentzek[8], Christian Brettschneider[9], Hans-Helmut König[9], Dagmar Weeg[10], Horst Bickel[10], Melanie Luppa[11], Francisca S. Rodriguez[2,11], Silka Dawn Freiesleben[12,13,39], Selin Erdogan[12,13,39], Chantal Unterfeld[12,14], Oliver Peters[12,13,39], Eike J.Spruth[12,15], Slawek Altenstein[12,15], Andrea Lohse[15], Josef Priller[10,12,15,16], Klaus Fliessbach[1,2], Xenia Kobeleva[2], Anja Schneider[1,2], Claudia Bartels[17], Björn H. Schott[17,18,37], Jens Wiltfang[17,18,19], Franziska Maier[20], Wenzel Glanz[21], Enise I. Incesoy[21,22], Michaela Butryn[21], Emrah Düzel[21,22], Katharina Buerger[23,24], Daniel Janowitz[24], Michael Ewers[23,24], Boris-Stephan Rauchmann[25], Robert Perneczky[23,25,26,27,38], Ingo Kilimann[28,29], Doreen Görß[29], Stefan Teipel[28,29], Christoph Laske[30,40], Matthias H. Munk[30,31], Annika Spottke[2,32], Nina Roy[2], Frederic Brosseron[2], Michael T. Heneka[1,2], Alfredo Ramirez[1,2,33,34,35], Renat Yakupov[21], Martin Scherer[4], Wolfgang Maier[1], Frank Jessen[2,20,33], Steffi G. Riedel-Heller[11]*, Michael Wagner[1,2] *

* equal contribution

1) Department of Neurodegenerative diseases and Geriatric Psychiatry, University Hospital Bonn, Bonn, Germany
2) German Center for Neurodegenerative Diseases (DZNE), Bonn, Germany
3) Developmental Psychology and Clinical Psychology of the Lifespan, University of Siegen, Siegen, Germany
4) Department of Primary Medical Care, Center for Psychosocial Medicine, University Medical Center Hamburg-Eppendorf, Hamburg, Germany
5) Institute of General Practice, Medical Faculty, University of Kiel, Kiel, Germany
6) Center for Information Management, Hannover Medical School, Hannover, Germany
7) Central Institute of Mental Health, Medical Faculty, Mannheim/Heidelberg University, Heidelberg, Germany
8) Institute of General Practice (ifam), Centre for Health and Society (chs), Medical Faculty, Heinrich Heine University, Düsseldorf
9) Department of Health Economics and Health Services Research, Hamburg Center for Health Economics, University Medical Center Hamburg-Eppendorf, Hamburg, Germany
10) School of Medicine, Technical University of Munich; Department of Psychiatry and Psychotherapy, Munich, Germany
11) Institute of Social Medicine, Occupational Health and Public Health (ISAP), Medical Faculty, University of Leipzig
12) German Center for Neurodegenerative Diseases (DZNE), Berlin, Germany

13) Department of Psychiatry, Campus Berlin-Buch, Charité – Universitätsmedizin Berlin, corporate member of Freie Universität Berlin, Humboldt-Universität zu Berlin, and Berlin Institute of Health (BIH)
14) Department of Psychiatry, Campus Benjamin Franklin, Charité – Universitätsmedizin Berlin, corporate member of Freie Universität Berlin, Humboldt-Universität zu Berlin, and Berlin Institute of Health (BIH)
15) Department of Psychiatry and Psychotherapy, Charité – Universitätsmedizin Berlin, Berlin, Germany
16) University of Edinburgh and UK DRI, Edinburgh, UK
17) Department of Psychiatry and Psychotherapy, University Medical Center Goettingen, University of Goettingen, Von-Siebold-Str. 5, 37075 Goettingen
18) German Center for Neurodegenerative Diseases (DZNE), Goettingen, Germany
19) Neurosciences and Signaling Group, Institute of Biomedicine (iBiMED), Department of Medical Sciences, University of Aveiro, Aveiro, Portugal
20) Department of Psychiatry, University of Cologne, Medical Faculty, Cologne, Germany
21) German Center for Neurodegenerative Diseases (DZNE), Magdeburg, Germany
22) Institute of Cognitive Neurology and Dementia Research (IKND), Otto-von-Guericke University, Magdeburg, Germany
23) German Center for Neurodegenerative Diseases (DZNE, Munich), Munich, Germany
24) Institute for Stroke and Dementia Research (ISD), University Hospital, LMU Munich, Munich, Germany
25) Department of Psychiatry and Psychotherapy, University Hospital, LMU Munich, Munich, Germany
26) Munich Cluster for Systems Neurology (SyNergy) Munich, Munich, Germany
27) Ageing Epidemiology Research Unit (AGE), School of Public Health, Imperial College London, London, UK
28) German Center for Neurodegenerative Diseases (DZNE), Rostock, Germany
29) Department of Psychosomatic Medicine, Rostock University Medical Center, Rostock, Germany
30) German Center for Neurodegenerative Diseases (DZNE), Tübingen, Germany
31) Department of Biology, Technische Universität Darmstadt, Darmstadt, Germany
32) Department of Neurology, University of Bonn, Bonn, Germany
33) Excellence Cluster on Cellular Stress Responses in Aging-Associated Diseases (CECAD), University of Cologne, Cologne, Germany
34) Division of Neurogenetics and Molecular Psychiatry, Department of Psychiatry and Psychotherapy, Faculty of Medicine, University Hospital Cologne, University of Cologne, Cologne, Germany
35) Department of Psychiatry & Glenn Biggs Institute for Alzheimer's and Neurodegenerative Diseases, San Antonio, Texas, USA
36) Department of Psychology and Centre for Ageing and Health (AgeCap), University of Gothenburg, Sweden
37) Leibniz Institute for Neurobiology, Magdeburg, Germany
38) Sheffield Institute for Translational Neuroscience (SITraN), University of Sheffield, Sheffield, UK

39) Memory Clinic and Dementia Prevention Center, Experimental and Clinical Research Center (ECRC), Berlin, Germany
40) Section for Dementia Research, Hertie Institute for Clinical Brain Research and Department of Psychiatry and Psychotherapy, University of Tübingen, Tübingen, Germany

Supplementary figure 1. Derivation of hypotheses on cognitive trajectories before dementia onset for brain maintenance (BM), brain reserve (BR) and cognitive reserve (CR)

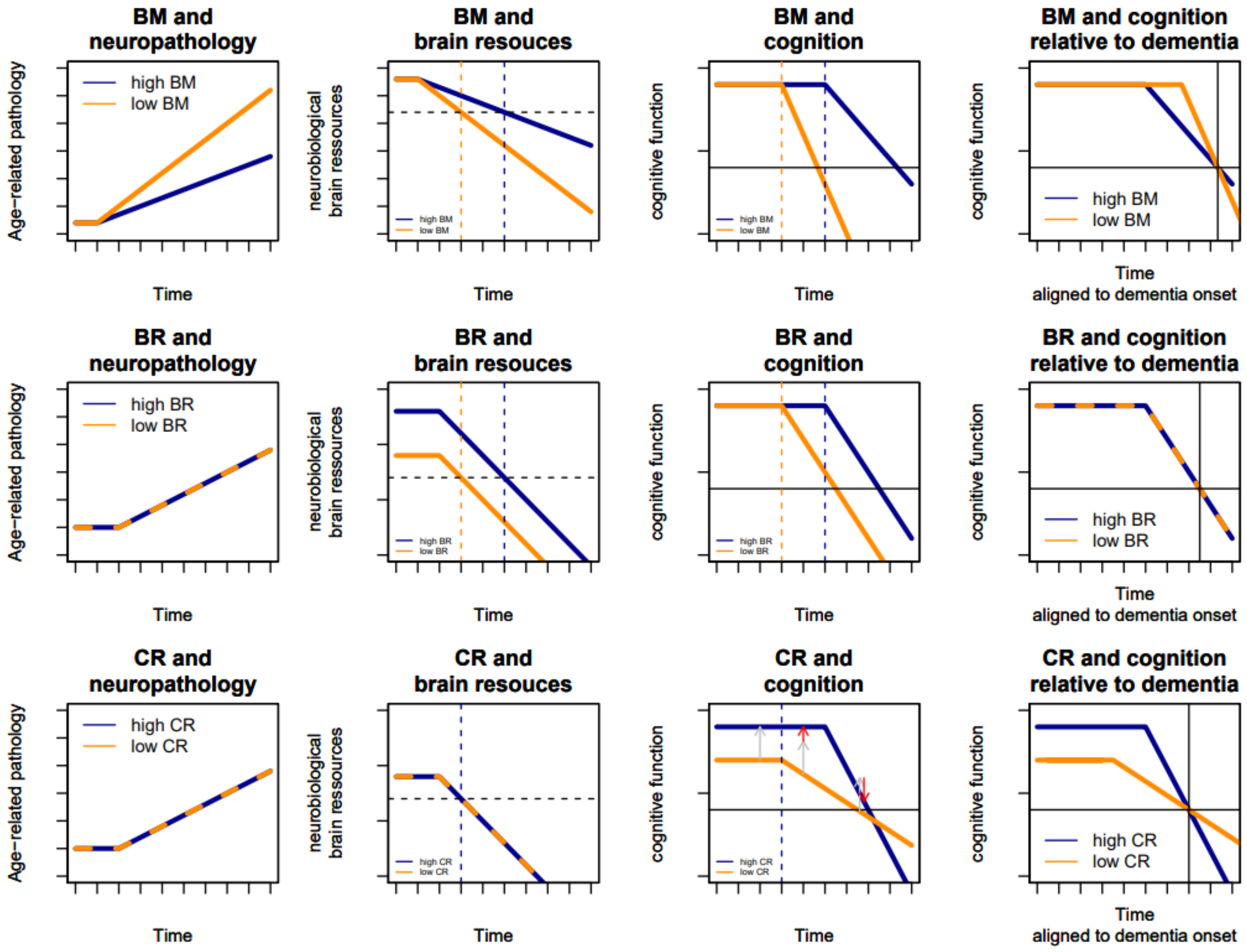

Notes. Plots in the first column (from left to right) indicate the development of age-related pathologies over time. Only BM theory would predict interindividual differences.

Plots in the second column (from left to right) illustrate how changes in age-related pathologies affect neurobiological brain resources.

We herein define brain resources as the quantitative amount of neurobiological capital of an individual that can be depleted by pathology before pathology shows any effect on cognitive function. Any interindividual differences in cognitive processes using or building upon these resources and any qualitative differences in brain features promoting cognitive processes are not considered as brain resources. The dotted horizontal line indicates the threshold for resource depletion that affects cognitive function. Dotted, vertical lines demark the time point at which the threshold is crossed.

According to BM theory, the rate of depletion should differ interindividually. According to BR, individuals differ regarding their brain resources before the onset of the development of age-related pathologies while CR theory would not predict any interindividual differences in brain resources (as defined above).

Plots in the third (from left to right) column illustrate how the depletion of brain resources translates into cognitive function. The black horizontal line indicates impairment corresponding to dementia. Dotted, vertical lines indicate demark the time point at which the threshold for resource depletion that affects cognitive function is crossed (same as in plots in the second column).

High BM (blue line) should be associated with a later onset (as indicated by the plot in the second column) and subsequently slower cognitive decline (as slopes should be identical to the rate of the accumulation of age-related pathologies shown in the plot in the first column).

Higher BR (blue line) should be associated with a later onset (see plot in second column) but subsequently no differences in the rate of decline (as slopes should be identical to the rate of the accumulation of age-related pathologies shown in the plot in the first column).

High CR (blue line) should be associated with a later onset of accelerated cognitive decline since the depletion of brain resources by pathology should be actively compensated by cognitive processes, thereby maintaining cognitive function. After a certain point of inflection, compensatory mechanisms can no longer maintain function and may even break down leading to an accelerated cognitive decline due to a catch up of the effect of pathology on cognitive function. Before the onset of the accumulation of age-related pathologies higher CR is expected to be associated with better cognitive function. Grey arrows (better cognitive function before onset of age-related pathologies) and red arrows (time-varying impact of CR) illustrate this relationship.

Plots in the fourth column (from left to right) show the very same lines for individuals with high (blue line) and low (orange line) BM, BR or CR, respectively, but they are shifted along the x-axis so that the threshold for dementia is crossed at the same time point. This is indicated by the vertical black line that crosses the horizontal black line (indicating the dementia threshold). These plots illustrate the hypotheses of each theory regarding the cognitive decline prior to the onset of dementia.

Supplementary text 1. Additional exclusion criteria of the German Center for Neurodegenerative Diseases Longitudinal Cognitive Impairment and Dementia Study (DELCODE) cohort

Medical conditions:

- current major depressive episode
- major psychiatric disorders either at baseline or in the past (e.g., psychotic disorder, bipolar disorder, substance abuse)
- neurodegenerative disorder other than AD
- vascular dementia
- history of stroke with residual clinical symptoms
- history of malignant disease
- severe or unstable medical condition
- clinically significant abnormalities in vitamin B12.

Prohibited drugs:

- chronic use of psychoactive compounds with sedative or anticholinergic effects,
- use of anti-dementia agents in SCD, amnestic MCI, and control subjects and in healthy siblings
- investigational drugs for treatment of dementia or cognitive impairment 1 month prior to entry and for the duration of the study

Supplementary text 2. O*Net job skills used to compute the occupational cognitive requirements score (OCRS)

4.A.2.b.6	Organizing, Planning, and Prioritizing Work
4.A.2.a.1	Judging the Qualities of Things, Services, or People
4.A.2.a.3	Evaluating Information to Determine Compliance with Standards
4.A.2.a.2	Processing Information
4.A.2.a.4	Analyzing Data or Information
4.A.2.b.1	Making Decisions and Solving Problems
4.A.2.b.2	Thinking Creatively
4.A.2.b.3	Updating and Using Relevant Knowledge
4.A.2.b.4	Developing Objectives and Strategies
4.A.2.b.5	Scheduling Work and Activities

Supplementary text 3. Methods for modeling cognitive decline in the DELOCDE cohort

To model cognitive change in DELCODE we used similar methods as described in the section Analysis of cognitive decline in AgeCoDe in the main manuscript. Latent process mixed models estimate a latent process as implemented in the R package" lcmm" were used to model cognitive change in the preclinical Alzheimer's disease cognitive composite (PACC5; see Papp, K. V, Rentz, D. M., Orlovsky, I., Sperling, R. A., and Mormino, E. C. (2017). Optimizing the preclinical Alzheimer's cognitive composite with semantic processing: The PACC5. Alzheimer's Dement. Transl. Res. Clin. Interv. 3, 668–677.). For the analysis, the average follow-up time was 3.727 years (SD=1.988).

The PACC5 was computed as the sum of z-scores of the FCSRT Free Recall and Total Recall, the MMSE, the Wechsler Memory Scale IV (WMS-IV) Logical Memory Story B delayed recall, the Symbol-Digit-Modalities Test, and the sum of the two category fluency tasks. The PACC5 was chosen as outcome measure due to its availability during follow-up. Factor scores used in the cross-sectional analyses of cognition were not available for longitudinal analyses.

Unequal interval scaling was accounted for using the beta cumulative distribution link function. Fixed effects of linear and quadratic time from baseline and the respective random effects were included and selected based on the BIC. Herein, the model including the quadratic term of time from baseline showed the best fit.

To assess the association of OCRS with cognitive decline, OCRS and its interactions with polynomials of time from baseline were modeled as fixed effects. Analyses were controlled for fixed effects of age, sex, years of education, and retirement status and (in case MRI markers were included) for intracranial volume, as well as their interactions with polynomials of time (e.g. time*age, time^2*age). To assess the interaction of OCRS with the respective pathological maker, three-way interaction of OCRS, maker, and the respective polynomials of time were included in the fixed effects. Multivariate Wald test were used to test the joint significance of these three-way interactions including polynomials of time. As for the cross-sectional analyses, we assessed the interaction of (polynomials of) time and the OCRS and CSF Aβ42/40-ratio, CSF pTau181, bilateral hippocampal volume, bilateral temporal cortex thickness, total grey matter volume and CSF total tau. MRI markers and intracranial volume were z-scaled in these analyses, no modifications were applied to the other variables in the model. Analyses were conducted in the whole sample, excluding patients with dementia of the Alzheimer's type (DAT) and, in addition, excluding patients with DAT and mild cognitive impairment (MCI).

Supplementary table 1. Sample description of the DELCODE cohort stratified by diagnosis

	Controls		DAT relatives		SCD		aMCI		DAT	
	M /N	SD / %	M /N	SD / %	M /N	SD / %	M /N	SD / %	M /N	SD / %
Age at baseline (Mean/SD)	69.71	5.34	66.47	4.56	71.45	6.04	73.04	5.7	75.36	6.79
Femal sex (N/%)	111	55.2	44	59.5	174	47.8	64	42.7	48	57.1
Years of education (Mean/SD)	14.77	2.76	14.47	2.76	14.88	2.98	14.01	3.15	13.07	3.1
MMSE at baseline (Mean/SD)	29.42	0.87	29.36	0.97	29.24	1.01	27.82	1.69	23.08	3.18
OCRS (Mean/SD)	3.97	0.82	3.88	0.86	4.07	0.78	3.8	0.87	3.65	0.76
Aβ42/40 (Mean/SD)	0.10	0.020	0.10	0.02	0.09	0.03	0.07	0.03	0.05	0.02
pTau181 (Mean/SD)	52.11	19.97	50.15	18.04	52.87	22.81	70.86	40.73	96.02	37.39
tTau (Mean/SD)	380.65	169.36	347.36	125.42	362.23	179.68	544.98	300.07	795.19	351.5
HCvol (Mean/SD)	3117.32	307.67	3102.11	311.21	3070.01	346.73	2776.63	417.45	2393.9	377.98
TempCor Thickness (Mean/SD)	2.82	0.10	2.83	0.08	2.78	0.12	2.69	0.15	2.54	0.19
total GM (Mean/SD)	600422.28	49409.42	596077.69	52130.98	600849.41	53759.29	575836.84	52734.5	550255.34	45733.49
Observation time (in years, Mean/SD)	3.58	1.75	2.82	1.51	2.84	1.62	2.52	1.72	1.74	1.46
MRI follow-up time (in years, Mean/SD)	1.04	0.07	1.06	0.11	1.04	0.09	1.02	0.08	1.03	0.06
CSF follow-up time (in years, Mean/SD)	2.83	0.95	2.60	0.91	2.52	0.92	2.52	0.83	2.46	0.71

Notes. Aβ42/40: CSF Aβ42/Aβ40 ratio; aMCI: amnestic cognitive impairment; APOE: Apolipoprotein E; CSF: cerebrospinal fluid; DAT: dementia of the Alzheimer's type; HCvol: average of left and right hippocampal volumes; N: sample size; OCRS: occupational cognitive requirement score; pTau181: CSF phospho-tau-181; SCD: subjective cognitive decline; SD: standard deviation; TempCor Thickness : temporal cortex thickness; total GM : total grey matter volume; tTau: CSF total Tau.

Supplementary table 2. Interaction analyses of OCRS with hippocampal volume and CSF biomarkers regarding cross-sectional memory function in DELCODE

whole sample				excluding DAT patients			
	Estimate	SE	p		Estimate	SE	p
Abeta-ratio (Abr; N=465)				Abeta-ratio (Abr; N=412)			
OCRS	0.036	0.044	4.14E-01	OCRS	0.047	0.037	2.09E-01
Abr	0.412	0.040	1.51E-22	Abr	0.244	0.035	2.07E-11
OCRS x Abr	-0.107	0.036	3.48E-03	OCRS x Abr	-0.095	0.032	2.84E-03
pTau181 (pTau; N=465)				pTau181 (pTau; N=412)			
OCRS	0.046	0.043	2.85E-01	OCRS	0.020	0.037	5.82E-01
ptTau	-0.457	0.043	2.59E-23	ptTau	-0.235	0.039	3.69E-09
OCRS x pTau	0.053	0.040	1.82E-01	OCRS x pTau	-0.015	0.030	6.12E-01
Hippocamapl volume (HCvol; N=793)				Hippocamapl volume (HCvol; N=718)			
OCRS	0.019	0.028	5.00E-01	OCRS	0.017	0.025	5.05E-01
HCvol	0.492	0.030	8.72E-53	HCvol	0.291	0.030	1.89E-21
OCRS x Hcvol	-0.079	0.025	1.42E-03	OCRS x Hcvol	-0.046	0.025	7.08E-02
Temporal cortex thickness (TCth; N=793)				Temporal cortex thickness (TCth; N=718)			
OCRS	0.046	0.029	1.11E-01	OCRS	0.022	0.025	3.74E-01
TCth	0.372	0.029	3.76E-35	TCth	0.203	0.028	1.35E-12
OCRS x TCth	-0.091	0.027	6.73E-04	OCRS x TCth	-0.038	0.027	1.59E-01
Total grey matter volume (tGM. N=793)				Total grey matter volume (tGM. N=718)			
OCRS	0.011	0.030	7.09E-01	OCRS	0.000	0.025	9.85E-01
tGM	0.462	0.040	2.98E-28	tGM	0.249	0.035	3.18E-12
OCRS x tGM	-0.051	0.027	6.07E-02	OCRS x tGM	-0.029	0.023	2.17E-01
total Tau (tTau; N=465)				total Tau (tTau; N=412)			
OCRS	0.016	0.042	7.04E-01	OCRS	0.013	0.037	7.14E-01
tTau	-0.477	0.039	1.43E-29	tTau	-0.294	0.040	1.14E-12
tTau x OCRS	0.015	0.037	6.91E-01	tTau x OCRS	-0.001	0.033	9.83E-01

Notes. Abeta-ratio: CSF Aβ42/Aβ40 ratio; CSF: cerebrospinal fluid; DAT: dementia of the Alzheimer's type; HCvol: average of left and right hippocampal volumes; OCRS: occupational cognitive requirement score; pTau181: CSF phospho-tau-181; SE: standard error; tTau: CSF total Tau.

Supplementary table 3. Interaction analyses of OCRS with hippocampal volume and CSF biomarkers regarding cross-sectional global cognitive function in DELCODE

whole sample				excluding DAT patients			
	Estimate	SE	p		Estimate	SE	p
Abeta-ratio (Abr; N=465)				Abeta-ratio (Abr; N=412)			
OCRS	0.055	0.035	1.15E-01	OCRS	0.068	0.031	3.04E-02
Abr	0.273	0.032	1.72E-16	Abr	0.160	0.029	7.24E-08
OCRS x Abr	-0.067	0.029	2.00E-02	OCRS x Abr	-0.058	0.026	2.80E-02
pTau181 (pTau; N=465)				pTau181 (pTau; N=412)			
OCRS	0.054	0.035	1.27E-01	OCRS	0.055	0.031	8.12E-02
ptTau	-0.288	0.033	1.14E-16	ptTau	-0.160	0.033	1.69E-06
OCRS x pTau	0.007	0.028	7.98E-01	OCRS x pTau	0.009	0.026	7.28E-01
Hippocamapl volume (HCvol; N=793)				Hippocamapl volume (HCvol; N=718)			
OCRS	0.054	0.025	2.74E-02	OCRS	0.057	0.023	1.26E-02
HCvol	0.341	0.026	3.57E-35	HCvol	0.194	0.027	1.38E-12
OCRS x Hcvol	-0.061	0.022	4.96E-03	OCRS x Hcvol	-0.039	0.023	9.09E-02
Temporal cortex thickness (TCth; N=793)				Temporal cortex thickness (TCth; N=718)			
OCRS	0.071	0.026	5.21E-03	OCRS	0.058	0.023	1.25E-02
TCth	0.279	0.025	2.03E-26	TCth	0.121	0.026	3.35E-06
OCRS x TCth	-0.079	0.024	8.28E-04	OCRS x TCth	-0.023	0.024	3.48E-01
Total grey matter volume (tGM. N=793)				Total grey matter volume (tGM. N=718)			
OCRS	0.035	0.026	1.70E-01	OCRS	0.035	0.023	1.18E-01
tGM	0.434	0.034	7.44E-34	tGM	0.266	0.032	2.34E-16
OCRS x tGM	-0.047	0.023	3.93E-02	OCRS x tGM	-0.034	0.021	1.03E-01
total Tau (tTau; N=465)				total Tau (tTau; N=412)			
OCRS	0.040	0.035	2.41E-01	OCRS	0.054	0.032	8.61E-02
tTau	-0.324	0.032	2.44E-21	tTau	-0.181	0.035	2.62E-07
tTau x OCRS	0.021	0.029	4.80E-01	tTau x OCRS	0.027	0.029	3.38E-01

Notes. Abeta-ratio: CSF Aβ42/Aβ40 ratio; CSF: cerebrospinal fluid; DAT: dementia of the Alzheimer's type; HCvol: average of left and right hippocampal volumes; OCRS: occupational cognitive requirement score; pTau181: CSF phospho-tau-181; SE: standard error; tTau: CSF total Tau.

Supplementary table 4. Interaction analyses of OCRS with hippocampal volume and CSF biomarkers regarding cross-sectional memory and global cognitive function in DELCODE excluding individuals with dementia of the Alzheimer's type (DAT) and mild cognitive impairment (MCI)

Memory function				Global cognitive function			
	Estimate	SE	p		Estimate	SE	p
Abeta-ratio (Abr; N=315)				Abeta-ratio (Abr; N=315)			
OCRS	-0.051	0.032	1.06E-01	OCRS	-0.008	0.030	7.89E-01
Abr	0.052	0.031	9.24E-02	Abr	0.031	0.030	2.91E-01
OCRS x Abr	0.030	0.029	2.97E-01	OCRS x Abr	0.050	0.028	7.40E-02
pTau181 (pTau; N=315)				pTau181 (pTau; N=315)			
OCRS	-0.058	0.030	5.73E-02	OCRS	0.003	0.030	9.26E-01
ptTau	-0.070	0.043	1.05E-01	ptTau	-0.050	0.042	2.33E-01
OCRS x pTau	-0.068	0.035	5.72E-02	OCRS x pTau	-0.034	0.034	3.23E-01
Hippocamapl volume (HCvol; N=598)				Hippocamapl volume (HCvol; N=589)			
OCRS	-0.039	0.024	9.57E-02	OCRS	0.017	0.023	4.53E-01
HCvol	0.077	0.029	8.59E-03	HCvol	0.054	0.029	6.02E-02
OCRS x Hcvol	0.039	0.026	1.37E-01	OCRS x Hcvol	0.026	0.026	3.11E-01
Temporal cortex thickness (TCth; N=598)				Temporal cortex thickness (TCth; N=589)			
OCRS	-0.030	0.024	1.99E-01	OCRS	0.019	0.023	4.07E-01
TCth	0.042	0.029	1.44E-01	TCth	0.003	0.028	9.02E-01
OCRS x TCth	0.017	0.029	5.65E-01	OCRS x TCth	0.029	0.029	3.16E-01
Total grey matter volume (tGM. N=598)				Total grey matter volume (tGM. N=589)			
OCRS	-0.029	0.022	1.92E-01	OCRS	0.021	0.022	3.28E-01
tGM	0.064	0.033	5.27E-02	tGM	0.113	0.032	4.22E-04
OCRS x tGM	-0.006	0.021	7.85E-01	OCRS x tGM	-0.010	0.020	6.17E-01
total Tau (tTau; N=315)				total Tau (tTau; N=315)			
OCRS	-0.066	0.032	4.04E-02	OCRS	0.002	0.031	9.39E-01
tTau	-0.086	0.044	5.18E-02	tTau	-0.054	0.043	2.02E-01
tTau x OCRS	-0.071	0.040	7.79E-02	tTau x OCRS	-0.025	0.039	5.16E-01

Notes. Abeta-ratio: CSF Aβ42/Aβ40 ratio; CSF: cerebrospinal fluid; DAT: dementia of the Alzheimer's type; HCvol: average of left and right hippocampal volumes; OCRS: occupational cognitive requirement score; pTau181: CSF phospho-tau-181; SE: standard error; tTau: CSF total Tau.

Supplementary table 5. Cross-sectional association of OCRS with CSF and MRI marker and interaction with age in DELCODE

whole sample				excluding DAT patients			
	Estimate	SE	p		Estimate	SE	p
Abeta-ratio (N=465)				Abeta-ratio (N=412)			
Main effect model							
OCRS	0.014	0.022	5.20E-01	OCRS	0.017	0.021	4.23E-01
Interaction model							
OCRS	0.017	0.022	4.34E-01	OCRS	0.021	0.022	3.40E-01
age	-0.148	0.022	2.34E-11	age	-0.121	0.023	2.11E-07
OCRS x age	0.020	0.018	2.88E-01	OCRS x age	0.014	0.019	4.63E-01
pTau181 (N=465)				pTau181 (N=412)			
Main effect model							
OCRS	-0.013	0.024	5.78E-01	OCRS	-0.010	0.023	6.52E-01
Interaction model							
OCRS	-0.017	0.024	4.76E-01	OCRS	-0.013	0.024	5.98E-01
age	0.162	0.024	2.60E-11	age	0.131	0.025	2.62E-07
OCRS x age	-0.027	0.020	1.77E-01	OCRS x age	-0.009	0.021	6.62E-01
Hippocamapl volume (N=794)				Hippocamapl volume (N=719)			
Main effect model							
OCRS	37.162	14.374	9.91E-03	OCRS	29.341	13.377	2.86E-02
Interaction model							
OCRS	36.693	14.461	1.14E-02	OCRS	25.410	13.605	6.22E-02
age	-204.963	14.955	1.89E-38	age	-173.182	14.445	2.93E-30
OCRS x age	-3.366	12.646	7.90E-01	OCRS x age	-20.886	12.226	8.80E-02
Temporal cortex thickness (N=794)				Temporal cortex thickness (N=719)			
Main effect model							
OCRS	0.010	0.005	4.82E-02	OCRS	0.006	0.005	2.22E-01
Interaction model							
OCRS	0.011	0.005	3.45E-02	OCRS	0.005	0.005	3.11E-01
age	-0.049	0.005	4.49E-19	age	-0.039	0.005	2.34E-13
OCRS x age	0.004	0.005	3.58E-01	OCRS x age	-0.004	0.004	4.05E-01
Total grey matter (N=794)				Total grey matter (N=719)			
Main effect model							
OCRS	5731.9	1490.9	1.31E-04	OCRS	5654.8	1503.1	1.83E-04
Interaction model							
OCRS	5737.9	1491.0	1.41E-04	OCRS	5537.0	1531.6	3.21E-04
age	-19141.9	1557.5	7.48E-32	age	-17246.2	1619.4	1.18E-24
OCRS x age	39.2	1313.1	9.76E-01	OCRS x age	-486.4	1369.9	7.23E-01
total Tau (N=465)				total Tau (N=412)			
Main effect model							
OCRS	-0.027	0.029	3.51E-01	OCRS	-0.029	0.028	3.09E-01
Interaction model							
OCRS	-0.031	0.029	2.97E-01	OCRS	-0.030	0.029	3.02E-01
age	0.182	0.029	9.16E-10	age	0.143	0.030	3.10E-06
OCRS x age	-0.026	0.025	2.86E-01	OCRS x age	-0.006	0.025	8.18E-01

Notes. Abeta-ratio: CSF Aβ42/Aβ40 ratio; CSF: cerebrospinal fluid; DAT: dementia of the Alzheimer's type; HCvol: average of left and right hippocampal volumes; OCRS: occupational cognitive requirement score; pTau181: CSF phospho-tau-181; SE: standard error; tTau: CSF total Tau.

Supplementary table 6. Association of OCRS with longitudinal levels and change in CSF and MRI markers in DELCODE

whole sample				excluding DAT patients			
	Estimate	SE	p		Estimate	SE	p
Abeta-ratio (N=189, Observations: 424)				Abeta-ratio (N=179, Observations: 403)			
Main effect model				Main effect model			
OCRS	0.012	0.032	7.03E-01	OCRS	0.011	0.031	7.24E-01
Interaction model				Interaction model			
OCRS x time	-0.001	0.005	8.53E-01	OCRS x time	-0.001	0.005	7.82E-01
pTau181 (N=189, Observations: 424)				pTau181 (N=179, Observations: 403)			
Main effect model				Main effect model			
OCRS	-0.019	0.035	5.76E-01	OCRS	-0.012	0.033	7.13E-01
Interaction model				Interaction model			
OCRS x time	-0.008	0.005	7.75E-02	OCRS x time	-0.008	0.005	9.76E-02
Hippocamapl volume (N=606, Observations: 1212)				Hippocamapl volume (N=567, Observations: 1134)			
Main effect model				Main effect model			
OCRS	37.767	16.433	2.02E-02	OCRS	31.055	15.807	4.67E-02
Interaction model				Interaction model			
OCRS x time	-0.870	5.033	8.61E-01	OCRS x time	0.563	5.103	9.11E-01
Temporal cortex thickness (N=606, Observations: 1212)				Temporal cortex thickness (N=567, Observations: 1134)			
Main effect model				Main effect model			
OCRS	0.007	0.005	1.91E-01	OCRS	0.006	0.005	2.63E-01
Interaction model				Interaction model			
OCRS x time	-0.001	0.002	4.57E-01	OCRS x time	-0.001	0.002	7.01E-01
Total grey matter (N=606, Observations: 1212)				Total grey matter (N=567, Observations: 1134)			
Main effect model				Main effect model			
OCRS	5164.0	1645.1	1.52E-03	OCRS	4859.0	1657.6	3.04E-03
Interaction model				Interaction model			
OCRS x time	-764.7	685.2	2.58E-01	OCRS x time	-777.8	727.2	2.78E-01
total Tau (N=189, Observations: 424)				total Tau (N=179, Observations: 403)			
Main effect model				Main effect model			
OCRS	-0.034	0.040	3.90E-01	OCRS	-0.027	0.038	4.64E-01
Interaction model				Interaction model			
OCRS x time	0.003	0.009	6.92E-01	OCRS x time	0.002	0.009	8.53E-01

Notes. Main effect model: Association of the OCRS with marker levels across all time points (no OCRS x time interaction included). Interaction model: Assessment of the association of the OCRS with change in marker levels. Abeta-ratio: CSF Aβ42/Aβ40 ratio; CSF: cerebrospinal fluid; DAT: dementia of the Alzheimer's type; HCvol: average of left and right hippocampal volumes; OCRS: occupational cognitive requirement score; pTau181: CSF phospho-tau-181; SE: standard error; tTau: CSF total Tau.

Supplementary table 7. Cross-sectional association of OCRS with CSF and MRI marker and interaction with age in DELCODE excluding individuals with dementia of the Alzheimer's type (DAT) and mild cognitive impairment (MCI)

	Estimate	SE	p
Abeta-ratio (N=315)			
Main effect model			
OCRS	-0.001	0.020	9.64E-01
Interaction model			
OCRS	-0.005	0.022	8.12E-01
age	-0.068	0.022	1.99E-03
OCRS x age	-0.010	0.019	6.04E-01
pTau181 (N=590)			
Main effect model			
OCRS	-0.008	0.025	7.47E-01
Interaction model			
OCRS	-0.012	0.026	6.55E-01
age	0.085	0.027	1.63E-03
OCRS x age	-0.010	0.024	6.80E-01
Hippocamapl volume (N=590)			
Main effect model			
OCRS	7.885	13.353	5.55E-01
Interaction model			
OCRS	0.709	13.933	9.59E-01
age	-151.962	14.668	3.61E-23
OCRS x age	-22.183	12.671	8.05E-02
Temporal cortex thickness (N=590)			
Main effect model			
OCRS	0.004	0.005	3.98E-01
Interaction model			
OCRS	0.002	0.005	6.53E-01
age	-0.038	0.005	4.68E-12
OCRS x age	-0.005	0.005	2.85E-01
Total grey matter (N=590)			
Main effect model			
OCRS	4209.836	1590.579	8.35E-03
Interaction model			
OCRS	3892.227	1665.037	1.98E-02
age	-16660.846	1737.443	2.69E-20
OCRS x age	-865.565	1508.982	5.66E-01
total Tau (N=315)			
Main effect model			
OCRS	-0.013	0.030	6.75E-01
Interaction model			
OCRS	-0.008	0.032	8.04E-01
age	0.081	0.033	1.42E-02
OCRS x age	0.014	0.029	6.31E-01

Notes. Abeta-ratio: CSF Aβ42/Aβ40 ratio; CSF: cerebrospinal fluid; DAT: dementia of the Alzheimer's type; HCvol: average of left and right hippocampal volumes; OCRS: occupational cognitive requirement score; pTau181: CSF phospho-tau-181; SE: standard error; tTau: CSF total Tau.

Supplementary table 8. Additional analyses of the association of OCRS with longitudinal levels and change in CSF and MRI markers in DELCODE

Analysis in the whole sample controlling for follow-up time per individual				Excluding MCI and DAT patients			
	Estimate	SE	p		Estimate	SE	p
Abeta-ratio (N=189. Observations: 424)				Abeta-ratio (N=146. Observations: 330)			
Main effect model				Main effect model			
OCRS	0.013	0.032	6.90E-01	OCRS	-0.022	0.033	5.03E-01
Interaction model				Interaction model			
OCRS x time	-0.002	0.005	6.50E-01	OCRS x time	-0.001	0.005	8.85E-01
pTau181 (N=189. Observations: 424)				pTau181 (N=146. Observations: 330)			
Main effect model				Main effect model			
OCRS	-0.019	0.035	5.85E-01	OCRS	-0.023	0.038	5.32E-01
Interaction model				Interaction model			
OCRS x time	-0.008	0.005	9.49E-02	OCRS x time	-0.010	0.006	6.95E-02
Hippocamapl volume (N=606. Observations: 1212)				Hippocamapl volume (N=469. Observations: 938)			
Main effect model				Main effect model			
OCRS	39.374	16.452	1.55E-02	OCRS	9.908	15.988	5.29E-01
Interaction model				Interaction model			
OCRS x time	-0.883	5.054	8.59E-01	OCRS x time	-2.439	5.325	6.41E-01
Temporal cortex thickness (N=606. Observations: 1212)				Temporal cortex thickness (N=469. Observations: 938)			
Main effect model				Main effect model			
OCRS	0.007	0.005	1.91E-01	OCRS	0.005	0.005	3.16E-01
Interaction model				Interaction model			
OCRS x time	-0.001	0.002	5.26E-01	OCRS x time	0.000	0.002	8.31E-01
Total grey matter (N=606. Observations: 1212)				Total grey matter (N=469. Observations: 938)			
Main effect model				Main effect model			
OCRS	5260.6	1649.8	1.27E-03	OCRS	3206.2	1766.8	6.52E-02
Interaction model				Interaction model			
OCRS x time	-775.3	687.7	2.53E-01	OCRS x time	-637.4	852.6	4.47E-01
total Tau (N=189. Observations: 424)				total Tau (N=146. Observations: 330)			
Main effect model				Main effect model			
OCRS	-0.031	0.040	4.22E-01	OCRS	-0.013	0.042	7.62E-01
Interaction model				Interaction model			
OCRS x time	0.006	0.009	4.73E-01	OCRS x time	-0.001	0.010	9.07E-01

Notes. Main effect model: Association of the OCRS with marker levels across all time points (no OCRS x time interaction included). Interaction model: Assessment of the association of the OCRS with change in marker levels. Abeta-ratio: CSF Aβ42/Aβ40 ratio; CSF: cerebrospinal fluid; DAT: dementia of the Alzheimer's type; HCvol: average of left and right hippocampal volumes; OCRS: occupational cognitive requirement score; pTau181: CSF phospho-tau-181; SE: standard error; tTau: CSF total Tau.

Supplementary table 9. Interaction of the OCRS and CSF AD biomarkers regarding longitudinal cognitive decline in the PACC5 in DELCODE

	whole sample (N=442, N[observations]=1592)				excluding DAT patients (N=408, N[observations]=1533)				excluding DAT & MCI patients (N=314, N(observations)=1268)			
**CSF Aβ42/Aβ40 ratio (Abeta-ratio)**												
	Est	SE	Wald	p	Est	SE	Wald	p	Est	SE	Wald	p
Abeta-ratio	77.101	20.252	3.807	1.4E-04	62.582	19.340	3.236	0.001	-15.918	20.145	-0.790	0.429
OCRS	0.886	0.460	1.927	0.054	0.872	0.446	1.954	0.051	-0.634	0.481	-1.319	0.187
Abeta-ratio x OCRS	-9.530	4.949	-1.926	0.054	-9.116	4.715	-1.933	0.053	5.614	4.786	1.173	0.241
time	1.849	1.233	1.500	0.134	1.660	1.384	1.200	0.230	2.452	1.367	1.794	0.073
time²	-0.089	0.244	-0.363	0.717	-0.056	0.281	-0.201	0.841	-0.193	0.258	-0.749	0.454
time x AB42	-2.429	9.072	-0.268	0.789	-2.529	11.342	-0.223	0.824	-14.606	10.805	-1.352	0.176
time² x AB42	0.544	1.831	0.297	0.766	0.501	2.373	0.211	0.833	2.730	2.095	1.303	0.193
time x OCRS	-0.085	0.212	-0.402	0.688	-0.055	0.275	-0.198	0.843	-0.347	0.262	-1.326	0.185
time² x OCRS	0.004	0.044	0.099	0.921	-4.4E-04	0.058	-0.007	0.994	0.046	0.052	0.881	0.378
time x Abeta-ratio x OCRS	2.181	2.200	0.991	0.321	1.899	2.767	0.686	0.493	4.360	2.582	1.689	0.091
time² x Abeta-ratio x OCRS	-0.291	0.441	-0.660	0.509	-0.248	0.577	-0.429	0.668	-0.707	0.502	-1.408	0.159
**Multivariate Wald Test**		Wald	df	p		Wald	df	p		Wald	df	p
time x Abeta-ratio x OCRS & time² x Abeta-ratio x OCRS		1.237	2	0.539		0.825	2	0.662		2.897	2	0.235
**CSF pTau181**												
	Est	SE	Wald	p	Est	SE	Wald	p	Est	SE	Wald	p
pTau181	-0.040	0.018	-2.259	0.024	-0.025	0.016	-1.520	0.129	0.029	0.024	1.236	0.216
OCRS	-0.026	0.317	-0.081	0.935	0.045	0.292	0.153	0.878	0.292	0.324	0.901	0.368
pTau181 x OCRS	0.002	0.004	0.457	0.648	0.001	0.004	0.217	0.828	-0.007	0.005	-1.361	0.173
time	1.750	1.012	1.729	0.084	1.539	1.019	1.511	0.131	1.504	1.069	1.407	0.160
time²	-0.064	0.205	-0.311	0.756	-0.038	0.206	-0.186	0.853	-0.021	0.210	-0.098	0.922
time x pTau181	-0.008	0.012	-0.688	0.491	-0.009	0.012	-0.737	0.461	-0.013	0.013	-1.003	0.316
time² x pTau181	0.002	0.002	0.766	0.444	0.002	0.002	0.830	0.406	0.003	0.002	1.052	0.293
time x OCRS	0.133	0.171	0.779	0.436	0.127	0.172	0.742	0.458	-0.009	0.185	-0.047	0.963
time² x OCRS	-0.016	0.034	-0.481	0.630	-0.015	0.034	-0.444	0.657	-0.002	0.036	-0.057	0.955
time x pTau181 x OCRS	-0.001	0.003	-0.271	0.786	-4.9E-04	0.003	-0.177	0.859	0.001	0.003	0.335	0.738
time² x pTau181 x OCRS	-4.0E-05	0.001	-0.074	0.941	-9.0E-05	0.001	-0.156	0.876	-3.1E-04	0.001	-0.531	0.596
**Multivariate Wald Test**		Wald	df	p		Wald	df	p		Wald	df	p
time x pTau181 x OCRS & time² x pTau181 x OCRS		0.561	2	0.755		0.520	2	0.771		0.384	2	0.825

Notes. Mixed models included fixed effects of age, sex, education, retirement, age x time, sex x time, education x time, retirement x time, age x time^2, sex x time^2, education x time^2, retirement x time^2 in addition to the fixed effects reported in this table.

Supplementary table 10. Interaction of the OCRS and MRI biomarkers (Hippocampal volume and temporal cortex thickness) regarding longitudinal cognitive decline in the PACC5 in DELCODE

	whole sample (N=761, N[observations]=2885)				excluding DAT patients (N=715, N[observations]=2811)				excluding DAT & MCI patients (N=590, N(observations)=2432)			
**Hippocampal volume (HCvol)**	Est	SE	Wald	p	Est	SE	Wald	p	Est	SE	Wald	p
HCvol	2.054	0.390	5.268	0.000	1.588	0.402	3.950	8.0E-05	0.117	0.418	0.281	0.779
OCRS	0.061	0.123	0.495	0.621	0.094	0.117	0.802	0.422	0.009	0.113	0.083	0.934
HCvol x OCRS	-0.189	0.096	-1.974	0.048	-0.172	0.097	-1.763	0.078	0.044	0.101	0.436	0.663
time	1.224	0.680	1.801	0.072	1.202	0.677	1.776	0.076	1.090	0.713	1.529	0.126
time^2	-0.131	0.129	-1.015	0.310	-0.132	0.129	-1.029	0.303	-0.095	0.136	-0.699	0.485
time x HCvol	0.339	0.206	1.648	0.099	0.305	0.203	1.504	0.133	0.165	0.245	0.674	0.500
time^2 x HCvol	-0.020	0.042	-0.480	0.631	-0.015	0.041	-0.379	0.704	3.2E-04	0.048	0.007	0.995
time x OCRS	0.038	0.055	0.695	0.487	0.041	0.055	0.749	0.454	0.006	0.059	0.101	0.919
time^2 x OCRS	-0.011	0.011	-1.085	0.278	-0.012	0.011	-1.136	0.256	-0.011	0.011	-0.938	0.348
time x HCvol x OCRS	-0.059	0.050	-1.183	0.237	-0.058	0.049	-1.183	0.237	-0.045	0.059	-0.768	0.442
time^2 x HCvol x OCRS	0.005	0.010	0.539	0.590	0.005	0.010	0.529	0.597	0.004	0.012	0.349	0.727
**Multivariate Wald Test**		Wald	df	p		Wald	df	p		Wald	df	p
time x HCvol x OCRS & time^2 x HCvol x OCRS		2.502	2	0.286		2.609	2	0.271		1.223	2	0.543
**Temporal Cortex Thickness (TCth)**	Est	SE	Wald	p	Est	SE	Wald	p	Est	SE	Wald	p
TCth	1.845	0.409	4.516	1.0E-05	0.901	0.398	2.264	0.024	-0.230	0.419	-0.549	0.583
OCRS	0.116	0.130	0.888	0.375	0.140	0.122	1.149	0.251	0.026	0.112	0.233	0.816
TCth x OCRS	-0.245	0.102	-2.392	0.017	-0.085	0.098	-0.866	0.387	0.081	0.101	0.797	0.425
time	0.839	0.639	1.312	0.189	0.747	0.657	1.136	0.256	0.474	0.678	0.699	0.485
time^2	-0.028	0.121	-0.234	0.815	-0.021	0.126	-0.166	0.868	0.044	0.130	0.342	0.733
time x TCth	-0.040	0.196	-0.205	0.838	-0.038	0.190	-0.202	0.840	-0.157	0.225	-0.696	0.486
time^2 x TCth	-0.013	0.041	-0.314	0.754	-0.011	0.039	-0.293	0.770	-0.005	0.043	-0.107	0.915
time x OCRS	0.048	0.055	0.874	0.382	0.050	0.055	0.909	0.363	0.006	0.055	0.104	0.917
time^2 x OCRS	-0.012	0.011	-1.102	0.270	-0.012	0.011	-1.089	0.276	-0.009	0.011	-0.800	0.424
time x TCth x OCRS	0.056	0.048	1.169	0.242	0.051	0.046	1.092	0.275	0.065	0.055	1.194	0.233
time^2 x TCth x OCRS	-0.001	0.010	-0.096	0.924	-0.001	0.009	-0.068	0.946	-0.001	0.010	-0.081	0.935
**Multivariate Wald Test**		Wald	df	p		Wald	df	p		Wald	df	p
time x TCth x OCRS & time^2 x TCth x OCRS		4.989	2	0.083		4.545	2	0.103		6.042	2	0.049

Notes. Mixed models included fixed effects of age, sex, education, retirement, intracranial volume, age x time, sex x time, education x time, retirement x time, intracranial volume x time, age x time^2, sex x time^2, education x time^2, retirement x time^2, intracranial volume x time^2 in addition to the fixed effects reported in this table.

Supplementary table 11. Interaction of the OCRS and biomarkers (CSF total Tau and total gray matter volume) regarding longitudinal cognitive decline in the PACC5 in DELCODE

	whole sample (tGM: N=761, N[observations]=2885) (total Tau: 442, N[observations]=1592)				excluding DAT patients (tGM: N=715, N[observations]=2811) (total Tau: N=408, N[observations]=1533)				excluding DAT & MCI patients (tGM: N=590, N(observations)=2432) (total Tau: N=314, N(observations)=1268)			
**Total gray matter volume (tGM)**	Est	SE	Wald	p	Est	SE	Wald	p	Est	SE	Wald	p
tGM	1.950	0.469	4.156	3.0E-05	1.756	0.445	3.948	8.0E-05	0.552	0.428	1.290	0.197
OCRS	0.058	0.128	0.451	0.652	0.048	0.119	0.402	0.688	-0.017	0.111	-0.156	0.876
tGM x OCRS	-0.132	0.112	-1.177	0.239	-0.176	0.106	-1.662	0.097	-0.039	0.100	-0.386	0.699
time	0.973	0.656	1.485	0.138	0.900	0.664	1.355	0.176	0.641	0.695	0.922	0.357
time²	-0.026	0.126	-0.210	0.834	-0.020	0.129	-0.156	0.876	0.037	0.135	0.272	0.785
time x tGM	0.587	0.206	2.854	0.004	0.576	0.205	2.804	0.005	0.506	0.218	2.315	0.021
time² x tGM	-0.083	0.041	-2.038	0.042	-0.081	0.041	-1.992	0.046	-0.068	0.043	-1.582	0.114
time x OCRS	0.023	0.055	0.412	0.680	0.023	0.055	0.409	0.682	-0.014	0.058	-0.251	0.802
time² x OCRS	-0.008	0.011	-0.746	0.456	-0.008	0.011	-0.734	0.463	-0.006	0.011	-0.569	0.569
time x tGM x OCRS	-0.093	0.049	-1.909	0.056	-0.096	0.049	-1.968	0.049	-0.098	0.052	-1.904	0.057
time² x tGM x OCRS	0.014	0.010	1.435	0.151	0.014	0.010	1.457	0.145	0.013	0.010	1.309	0.190
**Multivariate Wald Test**		df	Wald	p		df	Wald	p		df	Wald	p
time x tGM x OCRS & time² x tGM x OCRS		3.991	2	0.136		4.312	2	0.116		4.330	2	0.115
**CSF total Tau**	Est	SE	Wald	p	Est	SE	Wald	p	Est	SE	Wald	p
total Tau	-0.006	0.002	-2.663	0.008	-0.005	0.002	-2.045	0.041	0.003	0.003	1.034	0.301
OCRS	-0.134	0.301	-0.446	0.656	-0.092	0.280	-0.328	0.743	0.203	0.308	0.661	0.509
total Tau x OCRS	3.9E-04	0.001	0.667	0.505	3.5E-04	0.001	0.635	0.525	-0.001	0.001	-1.138	0.255
time	1.611	1.028	1.567	0.117	1.465	0.495	2.961	0.003	1.550	1.064	1.457	0.145
time²	-0.018	0.213	-0.086	0.931	-0.001	0.049	-0.022	0.983	-0.012	0.211	-0.056	0.955
time x total Tau	-2.4E-04	0.001	-0.174	0.862	-0.001	0.001	-0.508	0.611	-0.002	0.002	-0.880	0.379
time² x total Tau	2.0E-05	3.0E-04	0.077	0.939	1.0E-04	2.7E-04	0.370	0.711	2.6E-04	3.4E-04	0.775	0.438
time x OCRS	0.177	0.156	1.135	0.256	0.142	0.140	1.015	0.310	-0.012	0.175	-0.069	0.945
time² x OCRS	-0.031	0.032	-0.953	0.341	-0.025	0.028	-0.899	0.368	-0.007	0.034	-0.199	0.842
time x total Tau x OCRS	-2.2E-04	3.3E-04	-0.653	0.514	-1.1E-04	3.0E-04	-0.353	0.724	1.8E-04	4.0E-04	0.458	0.647
time² x total Tau x OCRS	3.0E-05	7.0E-05	0.422	0.673	1.0E-05	6.0E-05	0.194	0.846	-4.0E-05	8.0E-05	-0.477	0.633
**Multivariate Wald Test**		df	Wald	p		df	Wald	p		df	Wald	p
time x total Tau x OCRS & time² x total Tau x OCRS		0.552	2	0.759		0.174	2	0.916		0.232	2	0.890

Notes. Mixed models included fixed effects of age, sex, education, retirement, intracranial volume (for total gray matter only) , age x time, sex x time, education x time, retirement x time, intracranial volume x time, age x time^2, sex x time^2, education x time^2, retirement x time^2, intracranial volume x time^2 (for total gray matter only) in addition to the fixed effects reported in this table.

Supplementary table 12. Results of the latent process mixed models in AgeCoDe

Main effect model				
	Estimate	SE	Wald	p
APOE-ε4	-0.142	0.062	-2.294	2.18E-02
OCRS	0.125	0.038	3.291	1.00E-03
time	0.545	0.068	7.984	<1E-03
time²	-0.513	0.033	-15.409	<1E-03
time x APOE-ε4	-0.294	0.125	-2.354	1.86E-02
time² x APOE-ε4	-0.056	0.061	-0.916	3.60E-01
time x OCRS	0.029	0.084	0.349	7.27E-01
time² x OCRS	-0.004	0.043	-0.103	9.18E-01
Multivariate Wald Test		df	Wald	p
time x OCRS & time² x OCRS		2	0.314	8.55E-01
Interaction model				
	Estimate	SE	Wald	p
APOE-ε4	-0.135	0.063	-2.153	3.13E-02
OCRS	0.133	0.037	3.573	3.50E-04
APOE-ε4 x OCRS	-0.040	0.073	-0.551	5.82E-01
time	-0.053	0.008	-6.868	<1E-03
time²	-0.514	0.033	-15.393	<1E-03
time x APOE-ε4	-0.332	0.126	-2.625	8.66E-03
time² x APOE-ε4	-0.053	0.062	-0.844	3.98E-01
time x OCRS	-0.019	0.046	-0.426	6.70E-01
time² x OCRS	0.000	0.017	0.001	9.99E-01
time x APOE-ε4 x OCRS	0.231	0.143	1.617	1.06E-01
time² x APOE-ε4 x OCRS	-0.018	0.068	-0.270	7.87E-01
Multivariate Wald Test		df	Wald	p
time x APOE-ε4 x OCRS & time² x APOE-ε4 x OCRS		2	6.931	3.13E-02

Notes. Mixed models included fixed effects of age, sex, education, age x time, sex x time, education x time, age x time^2, sex x time^2, education x time^2 in addition to the fixed effects reported in this table.
Multivariate Wald tests were performed on interactions including time and time² as both terms jointly describe the cognitive trajectory. APOE: Apolipoprotein E; df: degrees of freedom; OCRS: occupational cognitive requirement score; p: p-value; SE: standard error; Wald: Wald test statistic.

Supplementary table 13. Results of the generalized additive mixed model of cognitive trajectories in the normalized MMSE relative to DAT onset in AgeCoDe

Main model (number of observations=2897)				
	edf	Ref.df	F	p
ti(time)	12.63	12.63	195.82	<2E-16
ti(OCRS)	1.00	1.00	9.48	2.09E-03
ti(time,OCRS)	6.61	6.61	3.26	3.84E-03
Complete observation time (number of observations=3111)				
	edf	Ref.df	F	p
ti(time)	13.90	13.90	192.22	<2E-16
ti(OCRS)	1.00	1.00	8.78	3.08E-03
ti(time,OCRS)	5.69	5.69	3.07	5.81E-03
Thin plate regression splines (number of observations=2897)				
	edf	Ref.df	F	p
ti(time)	12.92	12.92	190.98	< 2E-16
ti(OCRS)	1.00	1.00	9.89	1.68E-03
ti(time,OCRS)	6.27	6.27	2.93	5.27E-03

Notes. DAT: dementia of the Alzheimer's type; edf: effective degrees of freedom; F: F-value; MMSE: Mini-Mental-State-Examination; OCRS: occupational cognitive requirement score; p: p-value; Ref.df: reference degrees of freedom; SE: standard error; Wald: Wald test statistic.

Supplementary figure 2. Predicted normalized MMSE (normMMSE) and based on OCRS depending on time relative to DAT onset

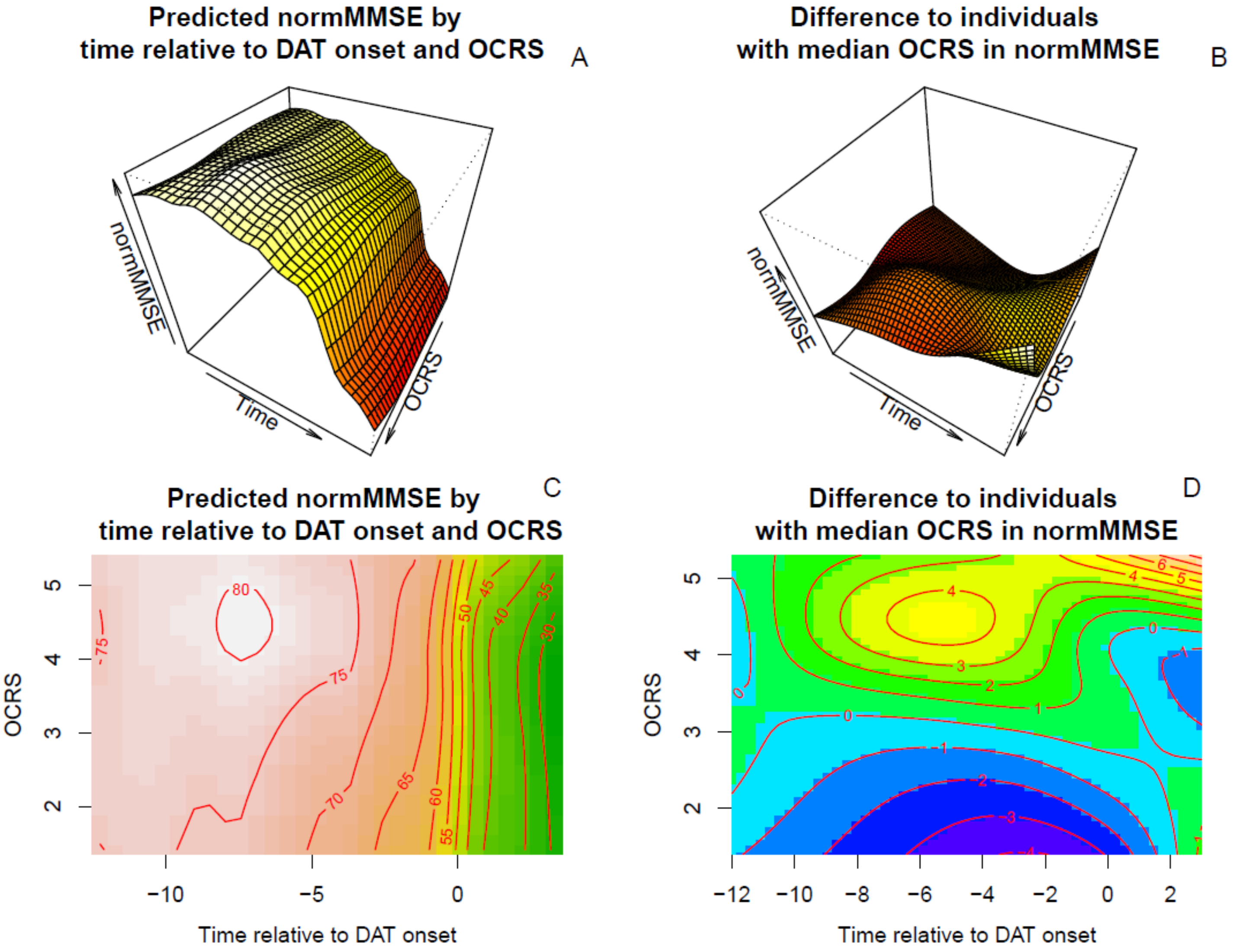

Notes. Panels on the left side (A,C) show predicted normalized MMSE (normMMSE; range: 0-100) values for time point relative to DAT onset (12 years before to 3 years after onset) and OCRS values. Same predicted values are shown as a perspective plot (A) or as a two dimensional color coded plot (C). In planel C, legends for predicted values are provided inside the figure along the red lines.

Panels on the right side (B,D) show the difference of predicted normMMSE values compared to individuals with median OCRS at different time points relative to DAT onset (12 years before to 3 years after onset). Differences are computed from the sum of OCRS smooth terms [ti(OCRS) in the mgcv package] and OCRS and time tensor product interaction terms [ti(time,OCRS) in the mgcv package]. Same predicted differences are shown as a perspective plot (B) or as a two dimensional color coded plot (D). In panel D, legends for predicted values are provided inside the figure along the red lines.
DAT: dementia of the Alzheimer's type; MMSE: Mini-Mental-State-Examination; normMMSE: normalized MMSE; OCRS: occupational cognitive requirement score.
